# Association of Body Mass Index and Age With Subsequent Breast Cancer Risk in Premenopausal Women

**DOI:** 10.1001/jamaoncol.2018.1771

**Published:** 2018-06-21

**Authors:** The Premenopausal Breast Cancer Collaborative Group, Minouk J. Schoemaker, Hazel B. Nichols, Lauren B. Wright, Mark N. Brook, Michael E. Jones, Katie M. O’Brien, Hans-Olov Adami, Laura Baglietto, Leslie Bernstein, Kimberly A. Bertrand, Marie-Christine Boutron-Ruault, Tonje Braaten, Yu Chen, Avonne E. Connor, Miren Dorronsoro, Laure Dossus, A. Heather Eliassen, Graham G. Giles, Susan E. Hankinson, Rudolf Kaaks, Timothy J. Key, Victoria A. Kirsh, Cari M. Kitahara, Woon-Puay Koh, Susanna C. Larsson, Martha S. Linet, Huiyan Ma, Giovanna Masala, Melissa A. Merritt, Roger L. Milne, Kim Overvad, Kotaro Ozasa, Julie R. Palmer, Petra H. Peeters, Elio Riboli, Thomas E. Rohan, Atsuko Sadakane, Malin Sund, Rulla M. Tamimi, Antonia Trichopoulou, Giske Ursin, Lars Vatten, Kala Visvanathan, Elisabete Weiderpass, Walter C. Willett, Alicja Wolk, Jian-Min Yuan, Anne Zeleniuch-Jacquotte, Dale P. Sandler, Anthony J. Swerdlow

**Affiliations:** 1 Division of Genetics and Epidemiology, The Institute of Cancer Research, London, United Kingdom; 2 Department of Epidemiology, University of North Carolina Gillings School of Global Public Health, Chapel Hill; 3 Biostatistics and Computational Biology Branch, National Institute of Environmental Health Sciences, National Institutes of Health, Durham, North Carolina; 4 Department of Medical Epidemiology and Biostatistics, Karolinska Institutet, Stockholm, Sweden; 5 Department of Epidemiology, Harvard T. H. Chan School of Public Health, Boston, Massachusetts; 6 Department of Clinical and Experimental Medicine, University of Pisa, Pisa, Italy; 7 Department of Population Sciences, Beckman Research Institute of City of Hope, Duarte, California; 8 Slone Epidemiology Center at Boston University, Boston, Massachusetts; 9 Institut National de la Santé et de la Recherche Medicale U1018, Institut Gustave Roussy, Centre d’Etude des Supports de Publicité, Université Paris-Saclay, Université Paris-Sud, and Université Versailles Saint-Quentin, Paris, France; 10 Department of Community Medicine, Faculty of Health Sciences, University of Tromsø, The Arctic University of Norway, Tromsø; 11 Department of Population Health and Perlmutter Cancer Center, New York University School of Medicine, New York City, New York; 12 Department of Epidemiology, Johns Hopkins Bloomberg School of Public Health, Baltimore, Maryland; 13 Public Health Direction and Biodonostia Research Institute and Centro de Investigación Biomédica en Red de Epidemiología y Salud Pública, Basque Regional Health Department, San Sebastian, Spain; 14 Nutrition and Metabolism Section, International Agency for Research on Cancer, Lyon, France; 15 Channing Division of Network Medicine, Department of Medicine, Brigham and Women’s Hospital, Harvard Medical School, Boston, Massachusetts; 16 Cancer Epidemiology and Intelligence Division, Cancer Council Victoria, Melbourne, Victoria, Australia; 17 Centre for Epidemiology and Biostatistics, School of Population and Global Health, University of Melbourne, Melbourne, Victoria, Australia; 18 Department of Biostatistics and Epidemiology, School of Public Health and Health Sciences, University of Massachusetts, Amherst; 19 Division of Cancer Epidemiology, German Cancer Research Center, Heidelberg, Germany; 20 Nuffield Department of Population Health, University of Oxford, Oxford, England; 21 Dalla Lana School of Public Health, University of Toronto, Toronto, Ontario, Canada; 22 Radiation Epidemiology Branch, Division of Cancer Epidemiology and Genetics, National Cancer Institute, Bethesda, Maryland; 23 Health Services and Systems Research, Duke-NUS (National University of Singapore) Medical School, Singapore; 24 Nutrional Epidemiology Unit, Karolinska Institutet, Institute of Environmental Medicine, Stockholm, Sweden; 25 Cancer Risk Factors and Life-Style Epidemiology Unit, Cancer Research and Prevention Institute, Florence, Italy; 26 School of Public Health, Imperial College, London, England; 27 Department of Public Health, Section for Epidemiology, Aarhus University, Aarhus, Denmark; 28 Radiation Effects Research Foundation, Hiroshima, Japan; 29 University Medical Center, Utrecht University, Utrecht, the Netherlands; 30 Department of Epidemiology and Population Health, Albert Einstein College of Medicine, Bronx, New York; 31 Department of Surgical and Perioperative Sciences, Umeå University, Umeå, Sweden; 32 Hellenic Health Foundation, Athens, Greece; 33 Cancer Registry of Norway, Institute of Population-Based Cancer Research, Oslo; 34 Institute of Basic Medical Sciences, University of Oslo, Oslo, Norway; 35 Department of Preventive Medicine, University of Southern California, Los Angeles; 36 Department of Public Health, Norwegian University of Science and Technology, Trondheim; 37 Genetic Epidemiology Group, Folkhälsan Research Center, Faculty of Medicine, University of Helsinki, Helsinki, Finland; 38 Department of Nutrition, Harvard T. H. Chan School of Public Health, Boston, Massachusetts; 39 University of Pittsburgh Graduate School of Public Health and UPMC Hillman Cancer Center, Pittsburgh, Pennsylvania; 40 Epidemiology Branch, National Institute of Environmental Health Sciences, National Institutes of Health, Durham, North Carolina; 41 Division of Breast Cancer Research, The Institute of Cancer Research, London, England

## Abstract

**Importance:**

The association between increasing body mass index (BMI; calculated as weight in kilograms divided by height in meters squared) and risk of breast cancer is unique in cancer epidemiology in that a crossover effect exists, with risk reduction before and risk increase after menopause. The inverse association with premenopausal breast cancer risk is poorly characterized but might be important in the understanding of breast cancer causation.

**Objective:**

To investigate the association of BMI with premenopausal breast cancer risk, in particular by age at BMI, attained age, risk factors for breast cancer, and tumor characteristics.

**Design, Setting, and Participants:**

This multicenter analysis used pooled individual-level data from 758 592 premenopausal women from 19 prospective cohorts to estimate hazard ratios (HRs) of premenopausal breast cancer in association with BMI from ages 18 through 54 years using Cox proportional hazards regression analysis. Median follow-up was 9.3 years (interquartile range, 4.9-13.5 years) per participant, with 13 082 incident cases of breast cancer. Participants were recruited from January 1, 1963, through December 31, 2013, and data were analyzed from September 1, 2013, through December 31, 2017.

**Exposures:**

Body mass index at ages 18 to 24, 25 to 34, 35 to 44, and 45 to 54 years.

**Main Outcomes and Measures:**

Invasive or in situ premenopausal breast cancer.

**Results:**

Among the 758 592 premenopausal women (median age, 40.6 years; interquartile range, 35.2-45.5 years) included in the analysis, inverse linear associations of BMI with breast cancer risk were found that were stronger for BMI at ages 18 to 24 years (HR per 5 kg/m^2^ [5.0-U] difference, 0.77; 95% CI, 0.73-0.80) than for BMI at ages 45 to 54 years (HR per 5.0-U difference, 0.88; 95% CI, 0.86-0.91). The inverse associations were observed even among nonoverweight women. There was a 4.2-fold risk gradient between the highest and lowest BMI categories (BMI≥35.0 vs <17.0) at ages 18 to 24 years (HR, 0.24; 95% CI, 0.14-0.40). Hazard ratios did not appreciably vary by attained age or between strata of other breast cancer risk factors. Associations were stronger for estrogen receptor–positive and/or progesterone receptor–positive than for hormone receptor–negative breast cancer for BMI at every age group (eg, for BMI at age 18 to 24 years: HR per 5.0-U difference for estrogen receptor–positive and progesterone receptor–positive tumors, 0.76 [95% CI, 0.70-0.81] vs hormone receptor–negative tumors, 0.85 [95% CI: 0.76-0.95]); BMI at ages 25 to 54 years was not consistently associated with triple-negative or hormone receptor–negative breast cancer overall.

**Conclusions and Relevance:**

The results of this study suggest that increased adiposity is associated with a reduced risk of premenopausal breast cancer at a greater magnitude than previously shown and across the entire distribution of BMI. The strongest associations of risk were observed for BMI in early adulthood. Understanding the biological mechanisms underlying these associations could have important preventive potential.

## Key Points

**Question:**

What is the association between body mass index and risk for breast cancer diagnosed before menopause?

**Finding:**

In this large pooled analysis of data on 758 592 premenopausal women, an inverse association of breast cancer risk with body mass index at 18 through 54 years of age was found, most strongly for body mass index at ages 18 through 24 years. The inverse association was strongest for hormone receptor–positive breast cancer, was evident across the entire distribution of body mass index, and did not materially vary by attained age or other characteristics of women.

**Meaning:**

Increased adiposity, in particular during early adulthood, may be associated with reductions in the risk of premenopausal breast cancer.

## Teaser

This pooled analysis of individual patient data investigates the association of body mass index with subsequent breast cancer risk, in particular by age, attained age, risk factors for breast cancer, and tumor characteristics, in premenopausal women.

## Introduction

Breast cancer is the most commonly diagnosed cancer among women worldwide, accounting for 25% of female cancer cases at all ages and a greater percentage among young women.^1^ Its complex etiology involves an unusually large range of factors, of which adiposity, often assessed as body mass index (BMI; calculated as weight in kilograms divided by height in meters squared), is important and appears to have opposing effects at premenopausal and postmenopausal ages.

Increased adiposity in childhood and before menopause has been reported to be inversely associated with the risk of breast cancer diagnosed at premenopausal and postmenopausal ages, whereas increased adiposity after menopause is positively associated with risk.^2,3,4,5,6,7,8,9,10,11^ However, because incidence rates are lower among premenopausal than postmenopausal women, individual studies have had limited ability to investigate the association of BMI with the risk of premenopausal breast cancer. Past studies have been case-control studies, with potential for bias, and most prospective studies have had modest numbers of cases, except for some recent studies in Asian^12,13^ or Jewish Israeli^14^ populations, but have not assessed risk at different ages, by tumor type, and by menopausal status at breast cancer diagnosis. Meta-analyses have aggregated studies that differed in age at BMI assessment, attained age of participants, and degree of adjustment for potential confounding, and results were not stratified by other risk factors.^2,3,4,5,15,16,17,18^ Some studies suggest that the association of premenopausal adiposity with risk varies by tumor characteristics,^2,7,17,18,19^ but larger studies are needed to provide stable estimates by hormone receptor status or intrinsic tumor subtype.

To undertake a more powerful and systematic analysis of the association of BMI with breast cancer risk in premenopausal women, we pooled individual-level data from 758 592 women, including 13 082 cases of breast cancer, from 19 prospective cohort studies using data from recruitment and follow-up questionnaires. We aimed to estimate the relative risk associated with BMI at different ages, age at breast cancer diagnosis, and breast cancer characteristics and to explore whether associations were modified by other risk factors for breast cancer.

## Methods

Information on the Premenopausal Breast Cancer Collaborative Group, a collaboration facilitated by the National Cancer Institute Cohort Consortium, has been published previously.^20^ In short, individual-level data were pooled from 19 prospective cohorts in North America (n = 9), Europe (n = 7), Asia (n = 2), and Australia (n = 1),^20^ with participants recruited from January 1, 1963, through December 31, 2013 and at least 100 breast cancer cases diagnosed before 55 years of age. Data were harmonized to a common template for 1 to 16 questionnaire rounds per study. Full details of the study cohorts are given in the eMethods in the Supplement. All contributing studies gained approval from institutional review boards and obtained consent from participants as per country-specific requirements.

We used information on self-reported or measured current weight and height from multiple questionnaire rounds and information reported on questionnaires about weight at ages before study entry to construct BMI within the age ranges of 18 to 24, 25 to 34, 35 to 44, and 45 to 54 years. We categorized BMI according to World Health Organization definitions.^21^ The analysis included all participants who were premenopausal, had no personal history of breast cancer at study entry, and had data for premenopausal BMI available.

All breast cancers included in this analysis occurred before menopause, with the main analytic end point being invasive or in situ premenopausal breast cancer overall. However, we also analyzed separately by invasive and in situ cancer, by immunohistochemistry data on estrogen receptor (ER) and progesterone receptor (PR) status, and by clinicopathologic surrogate definitions of intrinsic breast cancer subtype.

Hazard ratios (HRs) were obtained as estimates of the relative risk of breast cancer from Cox proportional hazards regression models^22^ with attained age as the underlying timescale. Follow-up for breast cancer started at study entry or the age after enrollment to which the BMI applied. Follow-up ended at breast cancer diagnosis, menopause (or hysterectomy), last follow-up, death, or age 55 years, whichever occurred first.

We first generated cohort-specific relative risk estimates and obtained a pooled estimate with a 2-stage model.^23^ Because no appreciable between-study heterogeneity was detected using the *I*^2^ statistic,^24^ the data were analyzed in a pooled data set. All presented analyses were adjusted for attained age (implicit in the Cox proportional hazards regression model), cohort, year of birth, age at menarche, age at first birth, time since last birth, parity, and family history of breast cancer. Covariate information was time updated, when possible, with information from follow-up questionnaires.

We analyzed BMI separately as categorical and continuous variables (per 5 kg/m^2^ [5.0-U] difference), assuming a log-linear dose-response association, the validity of which was checked using restricted cubic spline models.^25^ We tested for effect modification by other risk factors for breast cancer and by attained age using log-likelihood ratio tests.^26^ Analyses by breast tumor subtype were conducted using an augmentation method.^27^ This method allows estimation of separate risk factor associations for type-specific outcomes in a single model stratified on outcome type, obtained from a data set in which separate observations on each participant have been created for each outcome. Conducted sensitivity analyses are outlined in the eMethods in the Supplement. We used Stata, version 14.2 (StataCorp) for all analyses, with *P* < .05 indicating signficance.^28^

## Results

The analyses included 758 592 women (median age, 40.6 years; interquartile range, 35.2-45.5 years), among whom 13 082 in situ or invasive breast cancer cases occurred during 7.2 million premenopausal years of follow-up (median, 9.3 years; interquartile range, 4.9-13.5 years) (eTable 1 in the Supplement). Weight was provided at 1 to 14 (median, 2) follow-up rounds per study and was self-reported for 88.9% to 99.6% of weights, depending on age. Weight at ages 18 to 24 years was retrospectively reported for 96.9% and at later ages for less than 10% of women. Obesity (BMI≥30.0) was more common in women who were 45 years or older (11.1%), were nulliparous (12.4%), had an early menarche (17.0%), had a family history of breast cancer (12.8%), or were black (26.8%) (Table).

**Table.** Characteristics at Study Recruitment of Women Included in the Analyses

Characteristic	Participants, No. (BMI≥30.0, %)	Person-years of Follow-up, No. (%)^a^	Cancer Cases, No.
BMI^b^			
15.0-16.9	2843	29 293 (0.4)	53
17.0-18.4	20 245	221 540 (3.1)	442
18.5-24.9	499 146	4 901 964 (68.1)	9356
25.0-29.9	159 660	1 375 769 (19.1)	2257
30.0-34.9	51 413	442 769 (6.2)	678
35.0-49.9	25 285	227 485 (3.2)	296
Age at entry, y			
<25	17 627 (9.2)	211 220 (2.9)	74
25-34	167 744 (8.9)	2 585 847 (35.9)	3657
35-44	366 893 (10.1)	3 688 360 (51.2)	7404
≥45	206 328 (11.1)	713 394 (9.9)	1947
Age at menarche, y			
7-11	141 899 (17.0)	1 410 957 (19.6)	2712
12-13	391 822 (9.7)	3 849 467 (53.5)	7117
≥14	195 180 (6.2)	1 715 887 (23.8)	2871
Missing or no periods	29 691 (9.1)	222 509 (3.1)	382
Age at first birth, y			
<25	290 668 (11.2)	2 630 694 (36.5)	4186
25-34	273 023 (8.7)	2 583 161 (35.9)	5364
≥35	19 152 (9.5)	134 836 (1.9)	393
Nulliparous	121 920 (12.4)	1 311 508 (18.2)	2367
Age or whether parous unknown	53 829 (6.7)	538 621 (7.5)	772
No. of births^c^			
1	128 760 (10.4)	1 358 259 (25.2)	2583
2	252 325 (9.4)	2 213 928 (41.0)	4356
3	188 633 (10.8)	1 650 219 (30.6)	2900
Not known	18 341 (6.5)	178 049 (3.3)	172
Family history of breast cancer			
No	556 203 (10.1)	5 576 245 (77.5)	9478
Yes	75 299 (12.8)	625 576 (8.7)	2265
Not known	127 090 (8.4)	997 000 (13.8)	1339
Race/ethnicity			
White	419 130 (10.0)	4 437 300 (61.6)	8437
Black	52 903 (26.8)	586 734 (8.2)	1006
Asian	26 214 (2.5)	212 411 (3.0)	235
Other	7894 (13.7)	89 276 (1.2)	167
Not known	252 451 (7.5)	1 873 100 (26.0)	3237
Birth cohort			
Before 1930	23 849 (9.1)	98 669 (1.4)	178
1930-1939	66 110 (7.7)	655 904 (9.1)	1306
1940-1949	243 663 (8.4)	1 878 359 (26.1)	4088
1950-1959	282 307 (10.9)	2 983 715 (41.4)	5305
1960-1969	101 002 (13.7)	1 183 177 (16.4)	1970
1970-1979	33 904 (11.2)	341 589 (4.7)	219
1980 or later	7757 (8.1)	57 407 (0.8)	16
All	758 592 (10.1)	7 198 821 (100.0)	13 082

Abbreviation: BMI, body mass index (calculated as weight in kilograms divided by height in meters squared).

^a^ Percentages have been rounded and may not total 100. Owing to rounding, person-years may not sum to the total.

^b^ Indicates at study entry or, if missing, most recent retrospectively reported weight.

^c^ Includes parous only.

Increasing BMI was linearly associated with decreasing risk of breast cancer in the restricted cubic spline models (eFigures 1 and 2 in the Supplement), except that, for some ages and tumor types, there appeared to be a leveling of risk for underweight women (BMI<18.5) compared with those in the normal weight range (BMI, 18.5-24.9). We therefore restricted the linear modeling of BMI to values of 18.5 or greater.

**Figure 1.**
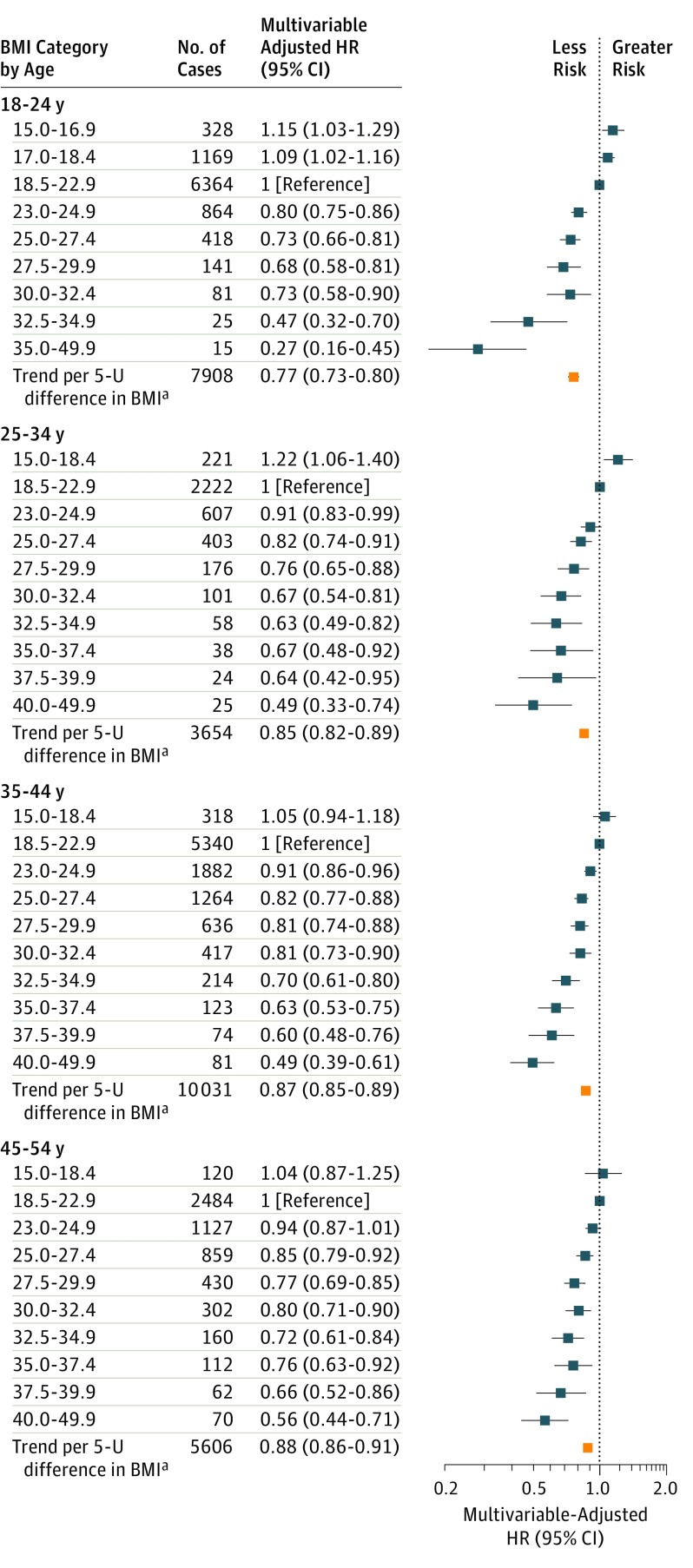
Relative Risk of Premenopausal Breast Cancer Associated With BMI Category, by Age at BMI Body mass index (BMI) is calculated as weight in kilograms divided by height in meters squared. Hazard ratios (HRs) were adjusted for attained age, cohort, year of birth, age at menarche, age at first birth, number of births, time since last birth, and family history of breast cancer. ^a^Represents linear trend per 5 kg/m^2^ (5.0-U) difference in BMI from 18.5 to 49.9.

Hazard ratios for breast cancer decreased with increasing BMI category (Figure 1), more for BMI at younger than older ages, with a risk reduction of 23% per 5.0-U difference (HR, 0.77; 95% CI, 0.73-0.80) for BMI at ages 18 to 24 years and 12% (HR, 0.88; 95% CI, 0.86-0.91) for BMI at 45 to 54 years. The risk gradient was 4.2-fold between the highest and lowest BMI categories (BMI≥35.0 vs <17.0) at ages 18 to 24 years (HR, 0.24; 95% CI, 0.14-0.40). Significant differences in relative risk were present even within the normal range of BMI (for 23.0-24.9 vs 18.5-22.9: HR, 0.80; 95% CI, 0.75-0.86). The HRs for BMI at ages 18 to 24 years remained statistically significant after additional adjustment for most recent BMI (HR per 5.0-U increase, 0.80; 95% CI, 0.76-0.84). We found no appreciable heterogeneity in the association between studies (eFigure 3A-D in the Supplement).

**Figure 2.**
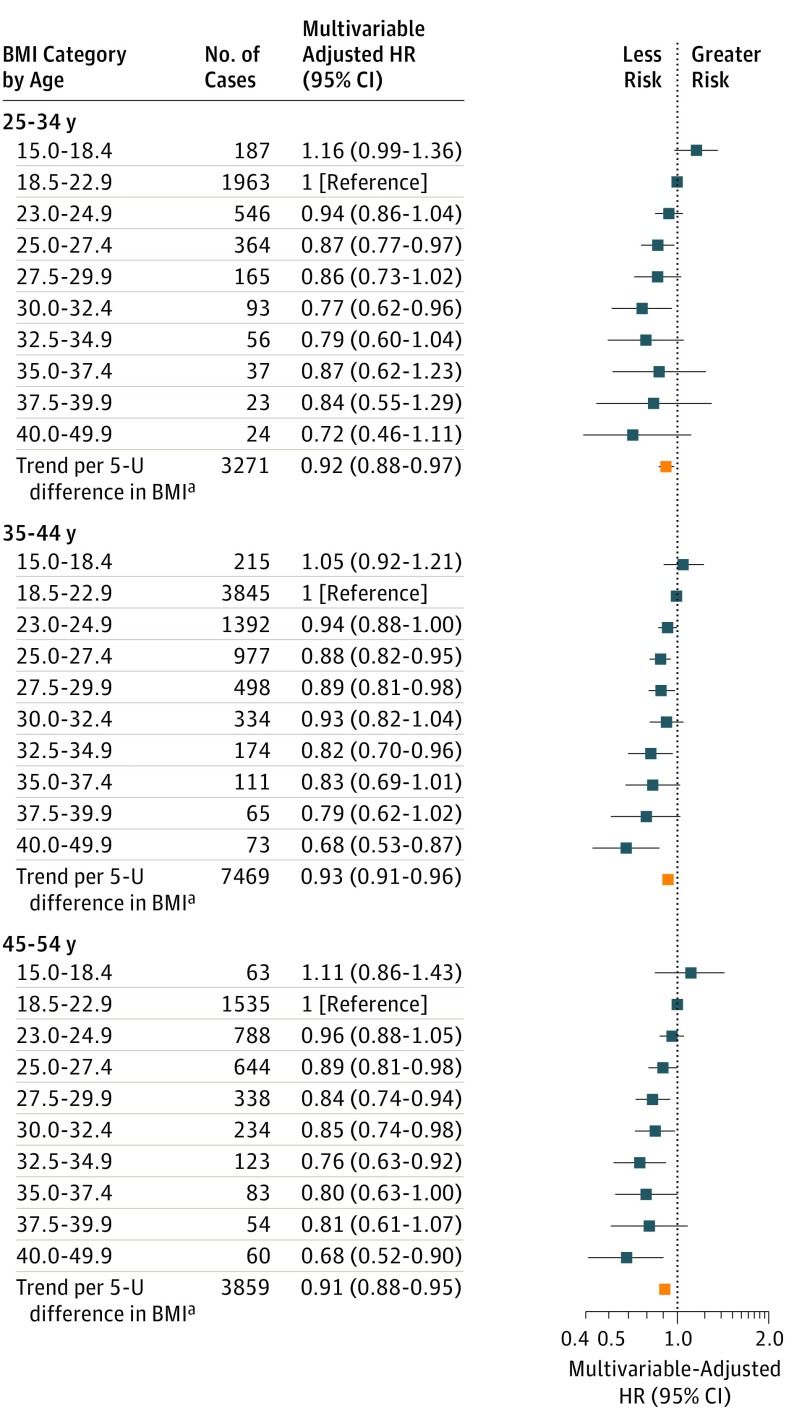
Relative Risk of Premenopausal Breast Cancer Associated With BMI and Adjusted for BMI at Ages 18 to 24 Years, by Age at BMI Body mass index (BMI) is calculated as weight in kilograms divided by height in meters squared. Hazard ratios (HRs) were adjusted for attained age, cohort, year of birth, age at menarche, age at first birth, number of births, time since last birth, and family history of breast cancer as well as BMI at ages 18 to 24 years. ^a^Represents linear trend per 5 kg/m^2^ (5.0-U) difference in BMI from 18.5 to 49.9.

Weight at ages 18 to 24 years was correlated with weights at older ages (*r* = 0.70 for ages 25-34, *r* = 0.63 for ages 35-44, and *r* = 0.55 for ages 45-54 years). When we adjusted the analyses of breast cancer risk in Figure 2 for BMI at ages 18 to 24 years, the HRs per 5.0-U increase were 0.92 (95% CI, 0.88-0.97) for BMI at ages 25 to 34 years, 0.93 (95% CI, 0.91-0.96) at ages 35 to 44 years, and 0.91 (95% CI, 0.88-0.95) at ages 45 to 54 years (Figure 2 and eTable 2 in the Supplement). The magnitude of the inverse associations was similar between categories of attained age to age 55 years (eFigure 4 in the Supplement).

Incident cases of breast cancer included 10 836 invasive and 2138 in situ breast cancers. Associations with risk per 5.0-U difference in BMI were significantly larger for in situ (HR, 0.76; 95% CI, 0.69-0.85) than for invasive breast cancer (HR, 0.88; 95% CI, 0.84-0.92) (*P* = .02 for interaction) for BMI at ages 25 to 34 years; difference in associations were also found for in situ (HR, 0.81; 95% CI, 0.76-0.86) and invasive breast cancer (HR, 0.88; 95% CI, 0.86-0.90; *P* = .01 for interaction) at 35 to 44 years (eTable 3 in the Supplement). The percentage of all breast cancers that were in situ did not appreciably vary by BMI, and women with higher BMI were more likely to have had a screening mammogram (eTable 4 in the Supplement).

**Figure 3.**
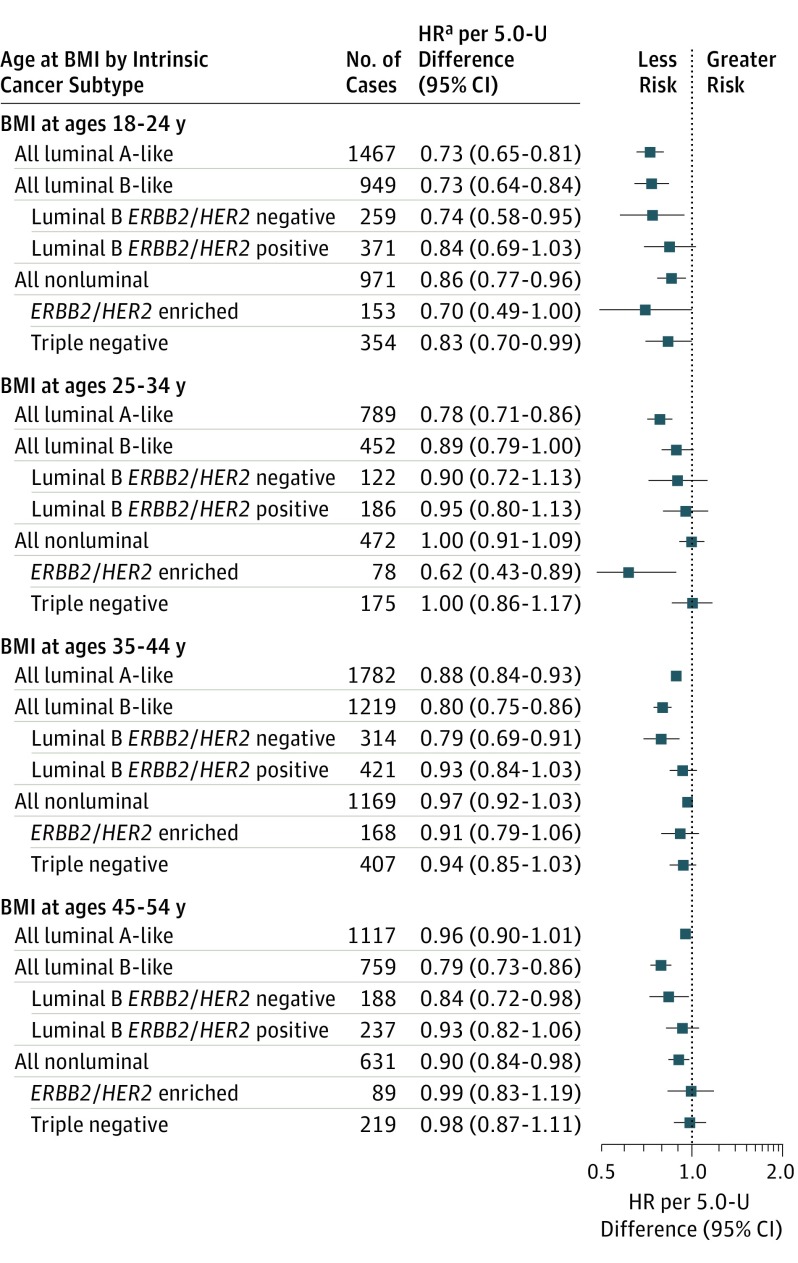
Relative Risk of Premenopausal Breast Cancer per 5 kg/m^2^ (5.0-U) Difference in BMI by Age at BMI and Breast Cancer Intrinsic Tumor Subtype Body mass index (BMI) is calculated as weight in kilograms divided by height in meters squared. The luminal A–like subtype includes estrogen receptor (ER)–positive, progesterone receptor (PR)–positive, and *ERBB2/HER2*-negative tumors; luminal B–subtype, all ER-positive and/or PR-positive tumors that are not luminal A–like (subtypes luminal B–like *ERBB2/HER2*-negative and luminal B–like *ERBB2/HER2*-positive); and nonluminal subtype, all ER-negative and PR-negative tumors, regardless of ERBB2/HER2 status (subtypes *ERBB2/HER2* enriched: ER-negative, PR-negative, and *ERBB2/HER2*-positive; triple-negative: ER-negative, PR-negative, and *ERBB2/HER2*-negative). ^a^Represents linear trend per 5.0-U of difference in BMI from 18.5 to 49.9 and are adjusted for attained age, cohort, year of birth, age at menarche, age at first birth, number of births, time since last birth, and family history of breast cancer. Estimates were obtained from 2 augmentation models.^27^ The first model included luminal A–like, luminal B–like, and nonluminal breast cancer as end points with tests for heterogeneity in effect by tumor type (for BMI at ages 18-24 years, *P* = .07; at ages 25-34 years, *P* = .002; at ages 35-44 years, *P* < .001; at ages 45-54, *P* < .001). Estimates for subtypes of luminal B–like and nonluminal breast cancer were obtained from a second model fitting luminal A–like, luminal B–like *ERBB2/HER2*-positive, luminal B–like *ERBB2/HER2*-negative, *ERBB2/HER2*-enriched, and triple-negative breast cancer as end points.

The ER and/or PR status was known for 7812 cases, 7002 (89.6%) of which were invasive. We did not observe consistent significant differences in HRs for ER-positive vs ER-negative or PR-positive vs PR-negative breast cancer (eTable 5 in the Supplement). When considering ER and PR jointly, ER-positive and/or PR-positive breast cancer showed stronger associations with BMI at ages 18 to 24 years (eg, for ER-positive and PR-positive, HR, 0.75; 95% CI, 0.70-0.81) than did hormone receptor–negative breast cancer; however, the association was still significant for ER-negative and PR-negative breast cancer (HR, 0.85; 95% CI, 0.76-0.95) (eFigure 5 in the Supplement). Body mass index at older ages was not associated with hormone receptor–negative breast cancer risk. In analyses by intrinsic breast cancer subtype (Figure 3), the nonluminal (ER-negative and PR-negative) subtype was inversely associated with BMI at ages 18 to 24 (HR, 0.86; 95% CI, 0.77-0.96) and 45 to 54 years (HR, 0.90; 95% CI, 0.84-0.98), but not with BMI at ages 25 to 34 and 35 to 44 years. No association of BMI at 25 years or older with triple-negative breast cancer or of BMI at 35 years or older with *ERBB2/HER2*-enriched breast cancer was found.

Hazard ratios per 5.0-U difference in BMI were not significantly different between strata of most risk factors for breast cancer, including race/ethnicity (for BMI at ages 18 to 24 years: HR for black women, 0.84 [95% CI, 0.76-0.93]; HR for Asian women, 0.69 [95% CI, 0.40-1.18]; and HR for white women, 0.73 [95% CI, 0.70-0.77]; *P* = .08 for interaction) (eFigure 6 and eTable 6 in the Supplement). Hazard ratios were greater for never users compared with than ever users of oral contraceptives (at baseline for most studies) for BMI at ages 18 to 24 years (HR, 0.68 [95% CI, 0.61-0.76] vs 0.79 [95% CI, 0.75-0.83]; *P* = .02 for interaction) and ages 35 to 44 (HR, 0.81 [95% CI, 0.77-0.86] vs 0.88 [95% CI, 0.86-0.91]; *P* = .009 for interaction). The HR was also greater for nulliparous than parous women (HR, 0.79 [95% CI, 0.73-0.87] vs 0.88 [95% CI, 0.84-0.93]; *P* = .03 for interaction) for BMI at ages 25 to 34 years. Results were not materially affected in the sensitivity analyses (eTables 7-9 in the Supplement) except for those shown in Figure 1.

## Discussion

In this large prospective analysis investigating the association between adiposity and breast cancer risk in premenopausal women, we analyzed relative risk by BMI in a larger number of categories than possible in previous studies, revealing a 4.2-fold risk gradient between women who were underweight vs obese at ages 18 to 24 years and a 1.9- to 2.5-fold risk gradient between these BMI categories at later ages. We demonstrated that the inverse associations apply to the entire distribution of BMI, with risk variations evident even among women in the nonoverweight range of BMI.

The observed inverse associations are unlikely to be attributable to bias, given that they were present in multiple studies and across strata of birth cohort and risk factors for breast cancer. Too few women died during follow-up (3.3% of women with a BMI≥35.0 vs 1.7% with BMI of 18.5-24.9) to explain the inverse associations for death as a competing risk. Our results are also supported as causal rather than artifactual by a mendelian randomization study^29^ reporting genetically predicted BMI to be inversely associated with breast cancer risk.

The stronger inverse associations of risk with BMI at younger than older ages suggest that adiposity in young adulthood or earlier, if adiposity at approximately 20 years of age is a proxy marker for adiposity in childhood, is the critical factor. No cohorts in our collaboration had information on BMI at younger than 18 years, but published analyses of subjective body size compared with peers at these ages^7,8,9^ have found strong inverse associations with premenopausal and postmenopausal breast cancer risk.

Our estimated 12% to 23% reduction in premenopausal breast cancer risk per 5.0-U difference in BMI depending on age is substantially stronger than that from meta-analyses,^3,4,5,15,16^ which have reported 5% to 9% reductions among women overall without analysis by age at BMI, and a study reporting a 10% reduction using measured BMI at ages 16 to 19 years.^14^ We observed that associations of BMI and breast cancer risk did not depend on attained age. We found no previous analyses by premenopausal attained age, but risk reductions with increased early-life BMI have been reported to continue after menopause.^7,8^ The associations of BMI with risk also did not appear to be appreciably modified by risk factors for breast cancer later in life, with the possible exception of nulliparity and oral contraceptive use.

The stronger associations of BMI at ages 25 to 44 years for in situ than for invasive breast cancer might reflect type-specific etiology or the association for in situ cancer being in part attributable to an association of body size with breast screening attendance. However, we found no evidence for this association, given that percentages of in situ cancer were similar across BMI groups.

No previous analyses of hormone receptor status–specific breast cancer by BMI assessed at different premenopausal ages have been performed, to our knowledge. We found that hormone receptor–positive breast cancer was associated with BMI at all ages and that hormone receptor–negative breast cancer overall was associated with BMI at ages 18 to 24 years, but not consistently associated with BMI at later ages; meta-analyses of ER- and PR-negative tumors^15,17^ have found no association, based on age at recruitment. The absence of an association of triple-negative breast cancer with BMI at 25 years or older in our analysis is contrary to previous reports^18,19^ indicating an increased risk of this tumor type with obesity; however, these reports were based on case-control studies^18^ and a pooled analysis of women younger than 50 years based on studies of mixed design^19^ and therefore are subject to potential biases that are of less concern in prospective cohorts. We observed that *ERBB2/HER2*-enriched breast cancer was associated with BMI at 35 years or younger but not at later ages; the Nurses’ Health Study, included in this analysis, previously reported a strong association with BMI at 18 years of age,^7^ but we are not aware of studies investigating the association with later premenopausal ages.

Obesity has many adverse effects on general health,^30^ and we do not advocate weight gain as a preventative measure against premenopausal breast cancer. However, understanding the mechanistic action underlying the inverse association of premenopausal adiposity with breast cancer risk could potentially identify modifiable pathways. Because the association with BMI at ages 18 to 24 years is significant for ER-positive and ER-negative tumors, hormonal and nonhormonal mechanisms might be involved. Breast tissue is particularly susceptible to carcinogens between menarche and first childbirth,^31^ and the extent of this susceptibility may be influenced by childhood adiposity. Increased adiposity has a strong association with early pubertal onset but also slower pubertal tempo,^32^ including slower peak growth,^33^ and rapid adolescent growth has been associated with increased breast cancer risk.^34^ The estrogenic effects of being overweight in childhood, when adipose tissue is the major site of estrogen release, have been proposed to induce early breast differentiation or to increase the expression of tumor suppressor genes.^35^ Being underweight during adolescence, in contrast, might result in immature differentiation due to lack of breast fat and/or low levels of ovarian hormones during breast development.^36^

Early-adulthood adiposity is associated with intermediate markers of breast cancer risk, such as benign breast disease,^37^ mammographic density,^38^ and insulinlike growth factor 1 levels.^39^ Greater mammographic density has a positive association with breast cancer risk,^40^ and a more endomorphic somatotype in childhood and early adulthood is associated with lower mammographic density throughout adulthood.^38^ In the Nurses’ Health Study,^41^ 82% of the association of BMI at 18 years of age with breast cancer risk was explained by mammographic density, and breast density may be an intermediate factor in the biological pathway for breast cancer development. However, the mechanism of how density affects risk is not well understood, and the amount of nondense (ie, fatty) tissue, with which BMI is strongly correlated, is also inversely associated with breast cancer risk, independently of percentage density.^40^ Early-life body size might also affect long-term insulinlike growth factor 1 levels implicated in breast cancer risk,^42^ given that plasma insulinlike growth factor 1 levels at ages 32 to 70 years were reported to be 14% lower in women who were overweight compared with those who were lean at age 18 years.^39^

The inverse association of BMI in premenopausal women 25 years or older with predominantly hormone receptor–positive rather than hormone receptor–negative breast cancer implies a hormonal mechanism. Estrogen synthesis, through the aromatase enzyme in subcutaneous fat, represents about 5% of total estradiol synthesis in premenopausal women, but with extreme obesity, negative feedback in the hypothalamic-pituitary-axis can lead to switch off of normal ovarian function and be reflected in amenorrhea.^43^ Irregular menstrual or fewer ovulatory cycles have therefore been suggested as possible explanations for the inverse association,^44^ but this suggestion has not been supported by studies that could adjust for menstrual cycle pattern.^9,10,11^ However, BMI-related differences in sex-hormone profile may contribute to the inverse association of BMI and breast cancer risk. A study^45^ reported that premenopausal women with higher BMI had lower estradiol, total testosterone, sex hormone–binding globulin, and progesterone levels but greater free testosterone levels than premenopausal women with lower BMI. Positive associations of premenopausal breast cancer risk have been reported with estradiol^46,47,48,49^ and testosterone levels,^46,47,48,50,51^ possibly stronger for ER-positive and/or PR-positive breast cancer,^47^ but studies have been inconsistent. Studies investigating endogenous progesterone levels,^46,47,49,50,51^ however, have not found associations with breast cancer risk. Circulating levels of leptin, a peptide hormone produced primarily by adipocytes that is overexpressed in breast cancer, were also associated with reduced premenopausal breast cancer risk in a prospective study, although not independently from BMI.^52^

### Strengths and Limitations

The strengths of this collaboration are its large number of cases, providing precise estimates of relative risk by age at adiposity and allowing for stratified and breast cancer subtype–specific analyses. All contributing studies were prospective, and most had multiple follow-up rounds, facilitating determination of menopausal status and time-updated covariate information.

However, the use of BMI has its limitations in that women with the same BMI can have different body fat distributions and overall body fat levels.^53^ Body mass index was recalled for early adulthood, but a good correlation (*r* = 0.87) has been reported between recalled and measured weight at 18 years of age.^54^ Current weight was usually self-reported, but in the Sister Study,^55^ 66% of women accurately reported their current weight within 1.35 kg, and underweight women tended to overreport and obese women tended to underreport, although rarely by more than 10%. If such misclassification applied to all studies, we might have slightly overestimated the trends but not to a sufficient degree to account for them fully. On the other hand, random misclassification would have led to attenuated effect sizes. Study-specific differences in method of assessment and cut points for hormone receptor and *ERBB2*/*HER2* status of breast cancer, given that these data were frequently obtained from medical records, may have led to differential classification of the tumor types between studies, potentially leading to underestimation of relative risks. More than 1000 breast cancer cases occurred in black women, allowing for stable estimation of relative risks in that population; results were similar to results from white women. However, we had insufficient statistical power to address the hypothesis that associations might be weaker or absent in Asian populations.^12,13,16,56^

## Conclusions

The results of our study suggest that increased BMI is inversely associated with the risk of breast cancer diagnosis before menopause, to a greater magnitude than suggested in previous analyses, and with the strongest associations for BMI at young ages. The association with BMI in early adulthood is universal across strata of other risk factors and across breast cancer subtypes. Understanding the biological mechanism underlying this association could have important implications for breast cancer prevention.

## Notes

Supplement. **eMethods.** Study Collaboration and Analysis **eTable 1.** Study Characteristics and Numbers of Premenopausal Women With Information on BMI by Cohort Study **eTable 2.** Relative Risk of Premenopausal Breast Cancer for All Participants and Those With Information on BMI at Ages 18-24 Years **eTable 3.** Relative Risk of Premenopausal Breast Cancer by Age at BMI and Invasiveness of Breast Cancer **eTable 4.** Characteristics of Breast Cancer Cases by Most Recent BMI Category Before Diagnosis **eTable 5.** Relative Risk of Premenopausal Breast Cancer Associated With BMI Category by Age at BMI and Estrogen or Progesterone Receptor Status of Breast Cancer **eTable 6.** Relative Risk of Premenopausal Breast Cancer per 5.0-U Difference in BMI by Age at BMI and Other Breast Cancer Risk Factors **eTable 7.** Relative Risk of Premenopausal Breast Cancer Associated With BMI Category by Age at BMI **eTable 8.** Relative Risk of Premenopausal Breast Cancer Associated With BMI Category at Ages 18 to 24 Years, Excluding Participants Contributing to Each Successive Cohort **eTable 9.** Relative Risk of Premenopausal Breast Cancer Associated With BMI Category at Ages 45 to 54 Years, Excluding Participants Contributing to Each Successive Cohort **eFigure 1.** Relative Risk of Premenopausal Breast Cancer Associated With BMI by Age at BMI **eFigure 2.** Relative Risk of Premenopausal Breast Cancer Associated With BMI at Ages 18 to 24 Years by Combined ER/PR Status of Breast Cancer **eFigure 3.** Forest Plot of Study-Specific Hazard Ratios of Premenopausal Breast Cancer by BMI Category and Age Category **eFigure 4.** Relative Risk of Premenopausal Breast Cancer per 5-U Difference in BMI by Age at BMI and Attained Age During Follow-up **eFigure 5.** Relative Risk of Premenopausal Breast Cancer per 5-U Difference in BMI by Age at BMI and Combined Estrogen and Progesterone Receptor Status of Breast Cancer **eFigure 6.** Relative Risk of Premenopausal Breast Cancer per 5-U Difference in BMI at Ages 18 to 24 Years by Selected Other Breast Cancer Risk Factors **eReferences.**

## References

[1] Torre LA, Islami F, Siegel RL, Ward EM, Jemal A Global cancer in women: burden and trends. Cancer Epidemiol Biomarkers Prev. 2017;26(4):-. 28223433 10.1158/1055-9965.EPI-16-0858

[2] Picon-Ruiz M, Morata-Tarifa C, Valle-Goffin JJ, Friedman ER, Slingerland JM Obesity and adverse breast cancer risk and outcome: mechanistic insights and strategies for intervention. CA Cancer J Clin. 2017;67(5):378-397. 28763097 10.3322/caac.21405 PMC5591063

[3] Renehan AG, Tyson M, Egger M, Heller RF, Zwahlen M Body-mass index and incidence of cancer: a systematic review and meta-analysis of prospective observational studies. Lancet. 2008;371(9612):569-578. 18280327 10.1016/S0140-6736(08)60269-X

[4] Cheraghi Z, Poorolajal J, Hashem T, Esmailnasab N, Doosti Irani A Effect of body mass index on breast cancer during premenopausal and postmenopausal periods: a meta-analysis. PLoS One. 2012;7(12):e51446. 23236502 10.1371/journal.pone.0051446 PMC3517558

[5] Kyrgiou M, Kalliala I, Markozannes G, Adiposity and cancer at major anatomical sites: umbrella review of the literature. BMJ. 2017;356:j477. 28246088 10.1136/bmj.j477 PMC5421437

[6] van den Brandt PA, Spiegelman D, Yaun SS, Pooled analysis of prospective cohort studies on height, weight, and breast cancer risk. Am J Epidemiol. 2000;152(6):514-527. 10997541 10.1093/aje/152.6.514

[7] Warner ET, Hu R, Collins LC, Height and body size in childhood, adolescence, and young adulthood and breast cancer risk according to molecular subtype in the Nurses’ Health Studies. Cancer Prev Res (Phila). 2016;9(9):732-738. 27590596 10.1158/1940-6207.CAPR-16-0085 PMC5012307

[8] Baer HJ, Tworoger SS, Hankinson SE, Willett WC Body fatness at young ages and risk of breast cancer throughout life. Am J Epidemiol. 2010;171(11):1183-1194. 20460303 10.1093/aje/kwq045 PMC2915489

[9] Oh H, Boeke CE, Tamimi RM, The interaction between early-life body size and physical activity on risk of breast cancer. Int J Cancer. 2015;137(3):571-581. 25335465 10.1002/ijc.29272 PMC4405425

[10] Michels KB, Terry KL, Willett WC Longitudinal study on the role of body size in premenopausal breast cancer. Arch Intern Med. 2006;166(21):2395-2402. 17130395 10.1001/archinte.166.21.2395

[11] Palmer JR, Adams-Campbell LL, Boggs DA, Wise LA, Rosenberg L A prospective study of body size and breast cancer in black women. Cancer Epidemiol Biomarkers Prev. 2007;16(9):1795-1802. 17855697 10.1158/1055-9965.EPI-07-0336

[12] Lee KR, Hwang IC, Han KD, Jung J, Seo MH Waist circumference and risk of breast cancer in Korean women: a nationwide cohort study. Int J Cancer. 2018;142(8):1554-1559. 29193045 10.1002/ijc.31180

[13] Chen MJ, Wu WY, Yen AM, Body mass index and breast cancer: analysis of a nation-wide population-based prospective cohort study on 1 393 985 Taiwanese women. Int J Obes (Lond). 2016;40(3):524-530. 26443343 10.1038/ijo.2015.205 PMC4786735

[14] Keinan-Boker L, Levine H, Derazne E, Molina-Hazan V, Kark JD Measured adolescent body mass index and adult breast cancer in a cohort of 951 480 women. Breast Cancer Res Treat. 2016;158(1):157-167. 27306419 10.1007/s10549-016-3860-6

[15] Munsell MF, Sprague BL, Berry DA, Chisholm G, Trentham-Dietz A Body mass index and breast cancer risk according to postmenopausal estrogen-progestin use and hormone receptor status. Epidemiol Rev. 2014;36:114-136. 24375928 10.1093/epirev/mxt010 PMC3873844

[16] Amadou A, Ferrari P, Muwonge R, Overweight, obesity and risk of premenopausal breast cancer according to ethnicity: a systematic review and dose-response meta-analysis. Obes Rev. 2013;14(8):665-678. 23615120 10.1111/obr.12028

[17] Suzuki R, Orsini N, Saji S, Key TJ, Wolk A Body weight and incidence of breast cancer defined by estrogen and progesterone receptor status—a meta-analysis. Int J Cancer. 2009;124(3):698-712. 18988226 10.1002/ijc.23943

[18] Pierobon M, Frankenfeld CL Obesity as a risk factor for triple-negative breast cancers: a systematic review and meta-analysis. Breast Cancer Res Treat. 2013;137(1):307-314. 23179600 10.1007/s10549-012-2339-3

[19] Yang XR, Chang-Claude J, Goode EL, Associations of breast cancer risk factors with tumor subtypes: a pooled analysis from the Breast Cancer Association Consortium studies. J Natl Cancer Inst. 2011;103(3):250-263. 21191117 10.1093/jnci/djq526 PMC3107570

[20] Nichols HB, Schoemaker MJ, Wright LB, The Premenopausal Breast Cancer Collaboration: a pooling project of studies participating in the National Cancer Institute Cohort Consortium. Cancer Epidemiol Biomarkers Prev. 2017;26(9):1360-1369. 28600297 10.1158/1055-9965.EPI-17-0246 PMC5581673

[21] WHO Expert Consultation Appropriate body-mass index for Asian populations and its implications for policy and intervention strategies. Lancet. 2004;363(9403):157-163. 14726171 10.1016/S0140-6736(03)15268-3

[22] Cox DR Regression models and life-tables. J R Stat Soc [Ser A]. 1972;34(2):187-220.

[23] Stukel TA, Demidenko E, Dykes J, Karagas MR Two-stage methods for the analysis of pooled data. Stat Med. 2001;20(14):2115-2130. 11439425 10.1002/sim.852

[24] Higgins JP, Thompson SG Quantifying heterogeneity in a meta-analysis. Stat Med. 2002;21(11):1539-1558. 12111919 10.1002/sim.1186

[25] Harrell F Regression Modeling Strategies: With Applications to Linear Models, Logistic Regression, and Survival Analysis. New York, NY: Springer; 2001.

[26] Buse A The likelihood ratio, Wald, and Lagrange multiplier tests—an expository note. Am Stat. 1982;36(3, pt 1):153-157.

[27] Lunn M, McNeil D Applying Cox regression to competing risks. Biometrics. 1995;51(2):524-532. 7662841

[28] StataCorp, ed. Stata Statistical Software: Release 14. College Station, Texas: StataCorp LP; 2015.

[29] Guo Y, Warren Andersen S, Shu XO, Genetically predicted body mass index and breast cancer risk: mendelian randomization analyses of data from 145 000 women of European descent. PLoS Med. 2016;13(8):e1002105. 27551723 10.1371/journal.pmed.1002105 PMC4995025

[30] Afshin A, Forouzanfar MH, Reitsma MB, ; GBD 2015 Obesity Collaborators Health effects of overweight and obesity in 195 countries over 25 years. N Engl J Med. 2017;377(1):13-27. 28604169 10.1056/NEJMoa1614362 PMC5477817

[31] Colditz GA, Frazier AL Models of breast cancer show that risk is set by events of early life: prevention efforts must shift focus. Cancer Epidemiol Biomarkers Prev. 1995;4(5):567-571. 7549816

[32] Martí-Henneberg C, Vizmanos B The duration of puberty in girls is related to the timing of its onset. J Pediatr. 1997;131(4):618-621. 9386670 10.1016/s0022-3476(97)70073-8

[33] Vizmanos B, Martí-Henneberg C, Clivillé R, Moreno A, Fernández-Ballart J Age of pubertal onset affects the intensity and duration of pubertal growth peak but not final height. Am J Hum Biol. 2001;13(3):409-416. 11460907 10.1002/ajhb.1065

[34] Berkey CS, Frazier AL, Gardner JD, Colditz GA Adolescence and breast carcinoma risk. Cancer. 1999;85(11):2400-2409. 10357411 10.1002/(sici)1097-0142(19990601)85:11<2400::aid-cncr15>3.0.co;2-o

[35] Hilakivi-Clarke L, Forsén T, Eriksson JG, Tallness and overweight during childhood have opposing effects on breast cancer risk. Br J Cancer. 2001;85(11):1680-1684. 11742488 10.1054/bjoc.2001.2109 PMC2363976

[36] Suzuki R, Saji S, Toi M Impact of body mass index on breast cancer in accordance with the life-stage of women. Front Oncol. 2012;2:123. 23061041 10.3389/fonc.2012.00123 PMC3463802

[37] Berkey CS, Rosner B, Tamimi RM, Body size from birth through adolescence in relation to risk of benign breast disease in young women. Breast Cancer Res Treat. 2017;162(1):139-149. 28062981 10.1007/s10549-016-4084-5 PMC5290089

[38] Yochum L, Tamimi RM, Hankinson SE Birthweight, early life body size and adult mammographic density: a review of epidemiologic studies. Cancer Causes Control. 2014;25(10):1247-1259. 25053404 10.1007/s10552-014-0432-0

[39] Poole EM, Tworoger SS, Hankinson SE, Schernhammer ES, Pollak MN, Baer HJ Body size in early life and adult levels of insulin-like growth factor 1 and insulin-like growth factor binding protein 3. Am J Epidemiol. 2011;174(6):642-651. 21828371 10.1093/aje/kwr123 PMC3166705

[40] Pettersson A, Tamimi RM Breast fat and breast cancer. Breast Cancer Res Treat. 2012;135(1):321-323. 22855239 10.1007/s10549-012-2186-2 PMC3764603

[41] Rice MS, Bertrand KA, VanderWeele TJ, Mammographic density and breast cancer risk: a mediation analysis. Breast Cancer Res. 2016;18(1):94. 27654859 10.1186/s13058-016-0750-0 PMC5031307

[42] Key TJ, Appleby PN, Reeves GK, Roddam AW; Endogenous Hormones and Breast Cancer Collaborative Group Insulin-like growth factor 1 (IGF1), IGF binding protein 3 (IGFBP3), and breast cancer risk: pooled individual data analysis of 17 prospective studies. Lancet Oncol. 2010;11(6):530-542. 20472501 10.1016/S1470-2045(10)70095-4 PMC3113287

[43] Dowsett M, Folkerd E Reduced progesterone levels explain the reduced risk of breast cancer in obese premenopausal women: a new hypothesis. Breast Cancer Res Treat. 2015;149(1):1-4. 25414027 10.1007/s10549-014-3211-4

[44] Key TJ, Pike MC The role of oestrogens and progestagens in the epidemiology and prevention of breast cancer. Eur J Cancer Clin Oncol. 1988;24(1):29-43. 3276531 10.1016/0277-5379(88)90173-3

[45] Tworoger SS, Eliassen AH, Missmer SA, Birthweight and body size throughout life in relation to sex hormones and prolactin concentrations in premenopausal women. Cancer Epidemiol Biomarkers Prev. 2006;15(12):2494-2501. 17164375 10.1158/1055-9965.EPI-06-0671

[46] Key TJ, Appleby PN, Reeves GK, ; Endogenous Hormones and Breast Cancer Collaborative Group Sex hormones and breast cancer risk in premenopausal women: collaborative reanalysis of seven prospective studies. Lancet Oncol. 2013;14(10):1009-1019. 23890780 10.1016/S1470-2045(13)70301-2 PMC4056766

[47] Eliassen AH, Missmer SA, Tworoger SS, Endogenous steroid hormone concentrations and risk of breast cancer among premenopausal women. J Natl Cancer Inst. 2006;98(19):1406-1415. 17018787 10.1093/jnci/djj376

[48] Walker K, Bratton DJ, Frost C Premenopausal endogenous oestrogen levels and breast cancer risk: a meta-analysis. Br J Cancer. 2011;105(9):1451-1457. 21915119 10.1038/bjc.2011.358 PMC3241538

[49] Fortner RT, Eliassen AH, Spiegelman D, Willett WC, Barbieri RL, Hankinson SE Premenopausal endogenous steroid hormones and breast cancer risk: results from the Nurses’ Health Study II. Breast Cancer Res. 2013;15(2):R19. 23497468 10.1186/bcr3394 PMC3672790

[50] Kaaks R, Tikk K, Sookthai D, Premenopausal serum sex hormone levels in relation to breast cancer risk, overall and by hormone receptor status—results from the EPIC cohort. Int J Cancer. 2014;134(8):1947-1957. 24155248 10.1002/ijc.28528

[51] Schernhammer ES, Sperati F, Razavi P, Endogenous sex steroids in premenopausal women and risk of breast cancer: the ORDET cohort. Breast Cancer Res. 2013;15(3):R46. 23777922 10.1186/bcr3438 PMC4053232

[52] Harris HR, Tworoger SS, Hankinson SE, Rosner BA, Michels KB Plasma leptin levels and risk of breast cancer in premenopausal women. Cancer Prev Res (Phila). 2011;4(9):1449-1456. 21680707 10.1158/1940-6207.CAPR-11-0125 PMC3168067

[53] Flegal KM, Shepherd JA, Looker AC, Comparisons of percentage body fat, body mass index, waist circumference, and waist-stature ratio in adults. Am J Clin Nutr. 2009;89(2):500-508. 19116329 10.3945/ajcn.2008.26847 PMC2647766

[54] Troy LM, Hunter DJ, Manson JE, Colditz GA, Stampfer MJ, Willett WC The validity of recalled weight among younger women. Int J Obes. 1995;19(8):570-572. 7489028

[55] Lin CJ, DeRoo LA, Jacobs SR, Sandler DP Accuracy and reliability of self-reported weight and height in the Sister Study. Public Health Nutr. 2012;15(6):989-999. 22152926 10.1017/S1368980011003193 PMC3511620

[56] Wada K, Nagata C, Tamakoshi A, ; Research Group for the Development and Evaluation of Cancer Prevention Strategies in Japan Body mass index and breast cancer risk in Japan: a pooled analysis of eight population-based cohort studies. Ann Oncol. 2014;25(2):519-524. 24412821 10.1093/annonc/mdt542

