# Supplementary material for: Association of Body Mass Index and Age With Subsequent Breast Cancer Risk in Premenopausal Women
Source: JAMA Oncol. 2018 Jun 21;4(11):e181771. doi: 10.1001/jamaoncol.2018.1771 (PMC6248078; doi:10.1001/jamaoncol.2018.1771)
Supplement: Supplement. — eMethods. Study Collaboration and Analysis eTable 1. Study Characteristics and Numbers of Premenopausal Women With Information on BMI by Cohort Study eTable 2. Relative Risk of Premenopausal Breast Cancer for All Participants and Those With Information on BMI at Ages 18-24 Years eTable 3. Relative Risk of Premenopausal Breast Cancer by Age at BMI and Invasiveness of Breast Cancer eTable 4. Characteristics of Breast Cancer Cases by Most Recent BMI Category Before Diagnosis eTable 5. Relative Risk of Premenopausal Breast Cancer Associated With BMI Category by Age at BMI and Estrogen or Progesterone Receptor Status of Breast Cancer eTable 6. Relative Risk of Premenopausal Breast Cancer per 5.0-U Difference in BMI by Age at BMI and Other Breast Cancer Risk Factors eTable 7. Relative Risk of Premenopausal Breast Cancer Associated With BMI Category by Age at BMI eTable 8. Relative Risk of Premenopausal Breast Cancer Associated With BMI Category at Ages 18 to 24 Years, Excluding Participants Contributing to Each Successive Cohort eTable 9. Relative Risk of Premenopausal Breast Cancer Associated With BMI Category at Ages 45 to 54 Years, Excluding Participants Contributing to Each Successive Cohort eFigure 1. Relative Risk of Premenopausal Breast Cancer Associated With BMI by Age at BMI eFigure 2. Relative Risk of Premenopausal Breast Cancer Associated With BMI at Ages 18 to 24 Years by Combined ER/PR Status of Breast Cancer eFigure 3. Forest Plot of Study-Specific Hazard Ratios of Premenopausal Breast Cancer by BMI Category and Age Category eFigure 4. Relative Risk of Premenopausal Breast Cancer per 5-U Difference in BMI by Age at BMI and Attained Age During Follow-up eFigure 5. Relative Risk of Premenopausal Breast Cancer per 5-U Difference in BMI by Age at BMI and Combined Estrogen and Progesterone Receptor Status of Breast Cancer eFigure 6. Relative Risk of Premenopausal Breast Cancer per 5-U Difference in BMI at Ages 18 to 24 Years by Selected Other Breast Cancer Risk F [file jamaoncol-4-e181771-s001.pdf]

Supplementary Online Content

The Premenopausal Breast Cancer Collaborative Group. Association of body mass index and age with premenopausal breast cancer risk in premenopausal women. *JAMA Oncol*. Published online June 21, 2018. doi:10.1001/jamaoncol.2018.1771

eMethods. Study Collaboration and Analysis

eTable 1. Study Characteristics and Numbers of Premenopausal Women With Information on BMI by Cohort Study

eTable 2. Relative Risk of Premenopausal Breast Cancer for All Participants and Those With Information on BMI at Ages 18-24 Years

eTable 3. Relative Risk of Premenopausal Breast Cancer by Age at BMI and Invasiveness of Breast Cancer

eTable 4. Characteristics of Breast Cancer Cases by Most Recent BMI Category Before Diagnosis

eTable 5. Relative Risk of Premenopausal Breast Cancer Associated With BMI Category by Age at BMI and Estrogen or Progesterone Receptor Status of Breast Cancer

eTable 6. Relative Risk of Premenopausal Breast Cancer per 5.0-U Difference in BMI by Age at BMI and Other Breast Cancer Risk Factors

eTable 7. Relative Risk of Premenopausal Breast Cancer Associated With BMI Category by Age at BMI

eTable 8. Relative Risk of Premenopausal Breast Cancer Associated With BMI Category at Ages 18 to 24 Years, Excluding Participants Contributing to Each Successive Cohort

eTable 9. Relative Risk of Premenopausal Breast Cancer Associated With BMI Category at Ages 45 to 54 Years, Excluding Participants Contributing to Each Successive Cohort

eFigure 1. Relative Risk of Premenopausal Breast Cancer Associated With BMI by Age at BMI

eFigure 2. Relative Risk of Premenopausal Breast Cancer Associated With BMI at Ages 18 to 24 Years by Combined ER/PR Status of Breast Cancer

eFigure 3. Forest Plot of Study-Specific Hazard Ratios of Premenopausal Breast Cancer by BMI Category and Age Category

eFigure 4. Relative Risk of Premenopausal Breast Cancer per 5-U Difference in BMI by Age at BMI and Attained Age During Follow-up

eFigure 5. Relative Risk of Premenopausal Breast Cancer per 5-U Difference in BMI by Age at BMI and Combined Estrogen and Progesterone Receptor Status of Breast Cancer

eFigure 6. Relative Risk of Premenopausal Breast Cancer per 5-U Difference in BMI at Ages 18 to 24 Years by Selected Other Breast Cancer Risk Factors

This supplementary material has been provided by the authors to give readers additional information about their work.

© 2018 American Medical Association. All rights reserved.

Body-mass index, age and premenopausal breast cancer risk:
A prospective analysis of 758,592 women

Supplementary methods and results

Table of contents

eMethods *3*

eResults
eTable 1: Study characteristics and numbers of premenopausal women with information on BMI, by cohort study *6*

eTable 2: Relative risk of premenopausal breast cancer in relation to BMI category, by age at BMI, for all subjects and subjects with information on BMI at age 18-24 years. *7*

eTable 3: Relative risk of premenopausal breast cancer in relation to BMI, by age at BMI and invasiveness of breast cancer *9*

eTable 4: Characteristics of breast cancer cases, by most recent BMI category prior to diagnosis *10*

eTable 5: Relative risk of premenopausal breast cancer in relation to BMI category, by age at BMI and oestrogen or progesterone receptor status of breast cancer *12*

eTable 6: Relative risk of premenopausal breast cancer per 5 kg/m^2 difference in BMI, by age at BMI and othe breast cancer risk factors *13*

eTable 7: Relative risk of premenopausal breast cancer in relation to BMI category, by age at BMI. For (1) all subjects included in main analysis (2) breast cancer with known ER, PR and HER2 status as endpoint (3) excluding subjects with weight assessed less than three years postpartum (4) strictly known premenopausal time only (5) excluding first two years of follow-up. *16*

eTable 8: Relative risk of premenopausal breast cancer in relation to BMI category at age 18-24 years, excluding subjects contributing to each successive cohort. *19*

eTable 9: Relative risk of premenopausal breast cancer in relation to BMI category at age 45-54 years, excluding subjects contributing to each successive cohort. *20*

eFigure 1: Relative risk of premenopausal breast cancer in relation to BMI relative to the reference category of 20 kg/m^2, by age at BMI *21*

eFigure 2: Relative risk of premenopausal breast cancer in relation to BMI at ages 18-24 years relative to the reference category of 20 kg/m^2, by combined ER/PR status of breast cancer *22*

eFigure 3a: Forest plot of study-specific hazard ratios of premenopausal breast cancer by BMI category, relative to BMI 18.5-24.9 kg/m^2, at age 18-24 years. *23*

eFigure 3b: Forest plot of study-specific hazard ratios of premenopausal breast cancer by BMI category, relative to BMI 18.5-24.9 kg/m^2, at age 25-34 years. *24*

eFigure 3c: Forest plot of study-specific hazard ratios of premenopausal breast cancer by BMI category, relative to BMI 18.5-24.9 kg/m^2, at age 35-44 years. *25*

eFigure 3d: Forest plot of study-specific hazard ratios of premenopausal breast cancer by BMI category, relative to BMI 18.5-24.9 kg/m^2, at age 45-54 years. *26*

© 2018 American Medical Association. All rights reserved.

eFigure 4: Relative risk of premenopausal breast cancer per 5 kg/m^2 difference in BMI, by age at BMI and attained age during follow-up *27*

eFigure 5: Relative risk of premenopausal breast cancer per 5 kg/m^2 difference in BMI, by age at BMI and combined oestrogen and progesterone receptor status of breast cancer *28*

eFigure 6: Relative risk of premenopausal breast cancer per 5 kg/m^2 difference in BMI at age 18-24 years, by selected other breast cancer risk factors *29*

eReferences *30*

© 2018 American Medical Association. All rights reserved.

eMethods

Cohort abbreviations

BWHS: Black Women’s Health Study

CLUE2: Campaign against Cancer and Heart Disease

CSDLH: Canadian Study of Diet, Lifestyle, and Health

CTS: California Teachers Study

E3N: Etude Epidémiologique auprès de femmes de la Mutuelle Générale de l'Education Nationale

EPIC: European Prospective Study into Cancer and Nutrition

GS: Generations Study

HUNT2: Helseundersøkelsen i Nord-Trøndelag

MCC: Melbourne Collaborative Cohort Study

NHS: Nurses’ Health Study

NHS2: Nurses’ Health Study 2

NOWAC: Norwegian Women and Cancer Study

NYUWHS: New York University Women’s Health Study

RERF: Radiation Effects Research Foundation Lifespan Study

SCHS: Singapore Chinese Health Study

SIS: Sister Study

SMC: Swedish Mammography Cohort

USRT: United States Radiologic Technologist Cohort

SWLHS: Sweden Women’s Lifestyle and Health Study.

© 2018 American Medical Association. All rights reserved.

The collaboration

Full details of the Premenopausal Breast Cancer Collaborative Group have been published elsewhere.[1] Individual-level data were pooled from 19 prospective cohorts with ≥100 breast cancer cases diagnosed before age 55 years, with the collaboration facilitated by the National Cancer Institute Cohort Consortium. Data were harmonised to a common template for 1-16 questionnaire rounds per study; all studies had at least two rounds except for the European Prospective Investigation into Cancer and Nutrition (EPIC) study, the Canadian Study of Diet, Lifestyle and Health (CSDLH), and HUNT2 for which only the baseline questionnaire was available. One study (CSDLH) provided data for a case-cohort subset; all the others provided data for the full cohort. Seventeen studies provided information on incident invasive and *in-situ* breast cancer and two (HUNT2 and Canadian Study of Diet, Lifestyle, and Health, CLDLH) on invasive breast cancer only.The pooled dataset was used to construct a dataset to investigate the endpoint of premenopausal breast cancer.

Derivation of age at menopause and premenopausal follow-up time

All cohorts collected information on menopausal status of participants at one or more questionnaire rounds. Participants were asked whether they had had any menstrual periods during the previous 6 or 12 months, depending on study, and/or whether they believed their periods had stopped permanently. Participants were asked about the age at their last period and the reason their periods stopped. We used this information to construct premenopausal follow-up time for analysis. Age at menopause was computed for each participant based on (i) reported age at menopause or, if age was missing, (ii) age first known postmenopausal if under age 50, (iii) age last known premenopausal if over age 50 or (iv) age 50 if no information was provided. When a hysterectomy was reported as reason for the menopause follow-up was censored at the reported age of the procedure. Since women with breast cancer often become postmenopausal due to their treatment and breast cancers diagnosed in the year of their menopause could be considered aetiologically premenopausal, we lagged reported menopausal ages (subjects under i) for all women by +1 year, i.e. the year during which they reported that they had become postmenopausal was analysed as premenopausal follow-up time. As a sensitivity analysis, we repeated the main analyses including only known premenopausal follow-up time up to the age at reported menopause (subjects under i) or, if age at menopause was missing, the age at the last questionnaire when the participant reported she was premenopausal (ii and iii).

Computation of BMI at various ages

We used data on current weight at the time of questionnaire completion and on recalled weight at ages before questionnaire completion, to construct variables for weight within the age ranges 18-24, 25-34, 35-44, 45-54 years. None of the studies had information to calculate BMI at ages younger than 18 years. Most weights at ages 18-24 years were retrospectively reported (most often for ages 18-21) but a minority were concurrently reported by subjects who were recruited at ages 18-24 years. At ages 25-34, 35-44 and 45-54, the majority of weights were concurrently reported. When weights were assessed on multiple occasions within an age category we used the earliest concurrent weight or otherwise the retrospectively reported weight relative to the youngest age within the age group. We recoded the following extreme values to missing based on visual inspection of histograms and percentile distributions: height (<130 or >195 cm), weight (<30, >200 kg), BMI (<15, >49 kg/m^2) and weights that arose from BMI values outside this range. In pooled analyses, BMI was categorised according to World Health Organization definitions[2] as severe/moderate thinness (<17 kg/m^2), mild thinness (17-18.5 kg/m^2), normal range (18.5-22.9 and 23.0-24.9 kg/m^2), overweight (25-27.4 and 27.5-29.9 kg/m^2), obese Class I (30-32.4 and 32.5-34.9 kg/m^2) and obese Class II/III (≥35 kg/m^2). Where numbers in the extreme categories were small or to obtain study-specific, stratum-specific or tumour type-specific estimates we combined categories to obtain stable estimates. For each age-specific BMI investigated, studies with less than 10 cases among subjects with known BMI were excluded from the model to improve convergence.

© 2018 American Medical Association. All rights reserved.

Clinicopathological surrogate definition of breast cancer intrinsic subtypes

Immunohistochemistry data on estrogen (ER) and progesterone receptor (PR) status, as well as data on Human epidermal growth factor receptor-2 (HER2) oncogene expression was collected from the centres. Given the absence of data on the proliferation marker KI-67, we adapted clinicopathological surrogate definitions of luminal A and luminal B-like intrinsic breast cancer subtypes proposed by the St Gallen Expert Consensus.[3] We classified all ER+PR+HER2- breast cancer as luminal A-like, ER+PR-HER2- and ER-PR+HER2- as luminal B-like HER2 negative, [ER+/PR+]HER2+ to luminal B-like HER2 positive, ER+PR- and ER-PR+ with HER2 status unknown as luminal B unclassified, ER+ or PR+ with other markers unknown as unclassified luminal, ER-PR-HER2- as triple-negative, ER-PR-HER2+ as HER2 enriched, ER- with PR unknown or PR- with ER unknown regardless of HER2 status as unclassified.

Statistical methods

Analyses were conducted using Stata 14.2 software[4]. BMI was analysed separately as a categorical and as a continuous variable (per 5 kg/m^2), assuming a log-linear dose-response relationship, the validity of which was checked using 5-knot restricted cubic spline models.[5] Hazard ratios (HR) as estimates of relative risk of breast cancer were obtained from Cox proportional hazards models[6] with attained age as the underlying time-scale. All analyses were conducted using Stata 14.2 software[4]. Pooled analyses were adjusted for attained age (implicit in the Cox model) and cohort (including country within EPIC). In multivariable-adjusted models we additionally adjusted for year of birth (<1930, 1930-9, 1940-9, 1950-9, 1960-9, 1970-9, to ≥1980), age at menarche (7-11, 12-13, ≥14 years, not known), age at first birth (<25, 25-34, ≥35 years, not known or not applicable), time since last birth (<5, 5-9, 10-14, 15-19, 20-24, 25-29, ≥30 years, not known or not applicable), parity (0, 1, 2, ≥3, parous but not known) and family history of breast cancer (yes, no, not known). Hazard ratios for breast cancer with respect to BMI were near-identical in age- and cohort-adjusted models compared with models additionally (fully) adjusted for other breast cancer risk factors. Fully adjusted models are therefore presented in the main paper. In models additionally adjusted for BMI at age 18-24, BMI at this age was coded as <18.5, 18.5-22.9, 23.0-24.9, 25.0-27.4, 27.5-29.9, ≥30 kg/m^2). Height was included as a continuous variable in models additionally adjusted for adult height. Covariate information was time-updated, where possible, with information from follow-up questionnaires for all pregnancy-related variables and family history of breast cancer. Subjects with missing covariate values were included in the analyses by fitting a category for the missing value.
In order to include the case-cohort study (CSDLH) in the pooled data set, we included Barlow weights[7] for CSDLH corresponding to a sampling fraction of 5.0 percent as an off-set in the model. We also applied Barlow weights with a sampling fraction of (effectively) 1.0 to all other cohorts using the stcasecoh command in Stata 14.2 software[4], which did not affect the results for those cohorts, but facilitated ease of obtaining results from a single pooled dataset.

Sensitivity analyses

In sensitivity analyses, we repeated the analyses (i) for BMI at ages 25 onwards adjusting for BMI at age 18-24 years (Figure 1) (ii) for BMI at ages 25 onwards restricted to individuals for whom BMI at ages 18-24 was also available (eTable 2) (iii) excluding subjects whose weight was recalled or reported less than three years postpartum (eTable 7) (iv) restricting follow-up to person-time that was known to be, rather than assumed to be, premenopausal (eTable 7) (v) excluding the first two years of follow-up (eTable 7) (vi) restricting the endpoint to breast cancer with information on all of ER, PR and HER2 status (eTable 7) (vii) excluding one cohort at the time (eTable 8) (viii) additional adjustment for adult height (not shown) (vix) comparing with and without adjustment for polycystic ovary syndrome (PCOS), for centres with data on PCOS (not shown).

© 2018 American Medical Association. All rights reserved.

eTable 1: Study characteristics and numbers of premenopausal women with information on BMI, by cohort study

Cohort	Country	Number of Subjects	Age at entry, median years	Age at entry, min years	Follow-up, median years	Number of Cases	BMI age 18-24 years		BMI age 25-34 years		BMI age 35-44 years		BMI age 45-54 years	
							Median BMI	Number of Subjects	Median BMI	Number of Subjects	Median BMI	Number of Subjects	Median BMI	Number of Subjects
BWHS	USA	45091	35.2	20.4	12	828	20.9	44482	25.3	21447	27.1	35361	28.4	25553
CLUE2	USA	4119	36.5	18.5	10.5	64	21	4110	23.3	1722	24.4	2879	25.6	2111
CSDLH (a)	Canada	1105	42.5	23.1	6.4	213	20.5	1099	22	156	22.9	552	23.2	381
CTS	USA	45708	41.2	22.2	8.8	641	20.8	45327	22.3	12107	23	23370	23.6	18184
E3N	France	58215	44.7	38.6	6.4	1245	-	0 (c)	-	0 (c)	21.4	30674	22	54722
EPIC (b)	Europe	94967	40.8	19.9	8.2	942	21.1	49045	22.2	15535	23.7	47837	24.8	27553
GS	UK	60916	38.4	18	6.4	655	21.6	59751	22.7	34825	23.8	47284	24.5	23865
HUNT2	Norway	16889	36.5	20.5	12	117	23.5	2090	24.1	5282	24.5	6423	25.3	3094
MCC	Australia	8097	45.4	31.1	4.6	86	20.8	7985	-	0 (c)	24.2	3720	24.9	6448
NHS	USA	94945	40.6	29.5	13.4	2334	20.9	76550	21.9	21372	22.5	65826	23.8	88173
NHS2	USA	83620	36.8	24.8	14.2	2515	20.6	82703	22.4	28926	23.2	82528	25.1	68850
NOWAC	Norway	75740	42.5	31.5	6.5	797	20.4	72012	22	3991	22.3	45472	23.5	44139
NYUWHS	USA	6649	43.5	31.5	7.5	241	-	0 (c)	22.1	85	22.9	4259	23.9	4544
RERF	Japan	11430	40.1	24.3	9.9	64	20.2	126	20.5	7599	21.5	6822	22.2	4721
SCHS	Singapore	9978	47.5	43.5	3.5	38	-	0 (c)	-	0 (c)	23.1	133	23.1	9857
SIS	USA	15314	46.3	35	4.5	397	-	0 (c)	-	0 (c)	23.4	15261	25.8	12844
SMC	Sweden	26110	44.8	38.4	6.7	332	20.1	15622	-	0 (c)	22.8	13572	23.6	15681
USRTC	USA	52682	34.4	22.2	12.2	911	20.6	40519	21.5	27083	22.9	42389	24.4	21376
WLHS	Sweden	47017	39.6	29.2	10.7	662	20.2	44319	22.2	12445	22.9	28834	23.8	17873
All		758592	40.6	18	9.3	13082	20.8	545740	22.3	192575	23.1	503196	24.0	449969

Abbreviations: BMI: Body-mass index; BWHS: Black Women's Health Study; CLUE2: Campaign against Cancer and Heart Disease; CSDLH: Canadian Study of Diet, Lifestyle, and Health; CTS: California Teachers Study; E3N: Etude Epidémiologique auprès de femmes de la Mutuelle Générale de l'Education Nationale; EPIC: European Prospective Study into Cancer and Nutrition; GS: Breast Cancer Now Generations Study; HUNT2: Helseundersøkelsen i Nord-Trøndelag; MCC: Melbourne Collaborative Cohort Study; NHS: Nurses' Health Study; NHS2: Nurses' Health Study 2; NOWAC: Norwegian Women and Cancer Study; NYUWHS: New York University Women's Health Study; RERF: Radiation Effects Research Foundation Lifespan Study; SCHS: Singapore Chinese Health Study; SIS: Sister Study; SMC: Swedish Mammography Cohort; USRT: United States Radiologic Technologist Cohort; SWLHS: Sweden Women's Lifestyle and Health Study. References for details of each cohort have previously been published [1] (a) case-cohort selected from a cohort of 28000 women recruited under age 55 (b) EPIC includes cohorts from 8 European countries (c) Data not collected at these age groups for these studies

© 2018 American Medical Association. All rights reserved.

eTable 2: Relative risk of premenopausal breast cancer in relation to BMI category, by age at BMI, for all subjects and subjects with information on BMI at age 18-24 years.

		All subjects		Subjects with BMI at age 18-24 years		
		Multivariable-adjusted		Multivariable-adjusted		Multivariable-adjusted plus BMI at age 18-24 years
Age at BMI	BMI category, kg/m^2	No. of cases	HR (95% CI) (a)	No. of cases	HR (95% CI) (a)	HR (95% CI) (b)
BMI at age 18-24 years						
	15.0-16.9	328	1.15 (1.03-1.29)	328	1.15 (1.03-1.29)	
	17.0-18.4	1169	1.09 (1.02-1.16)	1169	1.09 (1.02-1.16)	
	18.5-22.9	6364	1.00 (ref)	6364	1.00 (ref)	
	23.0-24.9	864	0.80 (0.75-0.86)	864	0.80 (0.75-0.86)	
	25.0-27.4	418	0.73 (0.66-0.81)	418	0.73 (0.66-0.81)	
	27.5-29.9	141	0.68 (0.58-0.81)	141	0.68 (0.58-0.81)	
	30.0-32.4	81	0.73 (0.58-0.90)	81	0.73 (0.58-0.90)	
	32.5-34.9	25	0.47 (0.32-0.70)	25	0.47 (0.32-0.70)	
	35.0-49.9	15	0.27 (0.16-0.45)	15	0.27 (0.16-0.45)	
	Trend per 5 kg/m^2 (c)	7908	0.77 (0.73-0.80)	7908	0.77 (0.73-0.80)	
BMI at age 25-34 years						
	15.0-18.4	221	1.22 (1.06-1.40)	187	1.21 (1.04-1.41)	1.16 (0.99-1.36)
	18.5-22.9	2222	1.00 (ref)	1963	1.00 (ref)	1.00 (ref)
	23.0-24.9	607	0.91 (0.83-0.99)	546	0.91 (0.83-1.00)	0.94 (0.86-1.04)
	25.0-27.4	403	0.82 (0.74-0.91)	364	0.81 (0.72-0.90)	0.87 (0.77-0.97)
	27.5-29.9	176	0.76 (0.65-0.88)	165	0.77 (0.66-0.91)	0.86 (0.73-1.02)
	30.0-32.4	101	0.67 (0.54-0.81)	93	0.66 (0.54-0.82)	0.77 (0.62-0.96)
	32.5-34.9	58	0.63 (0.49-0.82)	56	0.65 (0.50-0.85)	0.79 (0.60-1.04)
	35.0-37.4	38	0.67 (0.48-0.92)	37	0.68 (0.49-0.95)	0.87 (0.62-1.23)
	37.5-39.9	24	0.64 (0.42-0.95)	23	0.63 (0.42-0.95)	0.84 (0.55-1.29)
	40.0-49.9	25	0.49 (0.33-0.74)	24	0.49 (0.33-0.74)	0.72 (0.46-1.11)
	Trend per 5 kg/m^2 (c)	3654	0.85 (0.82-0.89)	3271	0.85 (0.82-0.89)	0.92 (0.88-0.97)
BMI at age 35-44 years						
	15.0-18.4	318	1.05 (0.94-1.18)	215	1.10 (0.96-1.26)	1.05 (0.92-1.21)
	18.5-22.9	5340	1.00 (ref)	3845	1.00 (ref)	1.00 (ref)

eTable 2: continued

© 2018 American Medical Association. All rights reserved.

		All subjects		Subjects with BMI at age 18-24 years		
		Multivariable-adjusted			Multivariable-adjusted	Multivariable-adjusted plus BMI at age 18-24 years
Age at BMI	BMI category, kg/m^2	No. of cases	HR (95% CI) (a)	No. of cases	HR (95% CI) (a)	HR (95% CI) (b)
	23.0-24.9	1882	0.91 (0.86-0.96)	1392	0.91 (0.85-0.97)	0.94 (0.88-1.00)
	25.0-27.4	1264	0.82 (0.77-0.88)	977	0.84 (0.78-0.90)	0.88 (0.82-0.95)
	27.5-29.9	636	0.81 (0.74-0.88)	498	0.82 (0.75-0.90)	0.89 (0.81-0.98)
	30.0-32.4	417	0.81 (0.73-0.90)	334	0.82 (0.74-0.92)	0.93 (0.82-1.04)
	32.5-34.9	214	0.70 (0.61-0.80)	174	0.71 (0.61-0.83)	0.82 (0.70-0.96)
	35.0-37.4	123	0.63 (0.53-0.75)	111	0.69 (0.57-0.84)	0.83 (0.69-1.01)
	37.5-39.9	74	0.60 (0.48-0.76)	65	0.64 (0.50-0.82)	0.79 (0.62-1.02)
	40.0-49.9	81	0.49 (0.39-0.61)	73	0.52 (0.41-0.65)	0.68 (0.53-0.87)
	Trend per 5 kg/m^2 (c)	10031	0.87 (0.85-0.89)	7469	0.88 (0.86-0.90)	0.93 (0.91-0.96)
BMI at age 45-54 years						
	15.0-18.4	120	1.04 (0.87-1.25)	63	1.12 (0.87-1.44)	1.11 (0.86-1.43)
	18.5-22.9	2484	1.00 (ref)	1535	1.00 (ref)	1.00 (ref)
	23.0-24.9	1127	0.94 (0.87-1.01)	788	0.94 (0.87-1.03)	0.96 (0.88-1.05)
	25.0-27.4	859	0.85 (0.79-0.92)	644	0.87 (0.79-0.95)	0.89 (0.81-0.98)
	27.5-29.9	430	0.77 (0.69-0.85)	338	0.80 (0.71-0.90)	0.84 (0.74-0.94)
	30.0-32.4	302	0.80 (0.71-0.90)	234	0.79 (0.69-0.91)	0.85 (0.74-0.98)
	32.5-34.9	160	0.72 (0.61-0.84)	123	0.69 (0.57-0.83)	0.76 (0.63-0.92)
	35.0-37.4	112	0.76 (0.63-0.92)	83	0.71 (0.57-0.89)	0.80 (0.63-1.00)
	37.5-39.9	62	0.66 (0.52-0.86)	54	0.71 (0.54-0.93)	0.81 (0.61-1.07)
	40.0-49.9	70	0.56 (0.44-0.71)	60	0.57 (0.44-0.74)	0.68 (0.52-0.90)
	Trend per 5 kg/m^2 (c)	5606	0.88 (0.86-0.91)	3859	0.88 (0.85-0.91)	0.91 (0.88-0.95)

Abbreviations: BMI, Body-mass Index; CI, confidence interval; HR, hazard ratio

(a) HRs adjusted for attained age, cohort, year of birth, age at menarche, age at first birth, number of births, time since last birth and family history of breast cancer
(b) Additionally adjusted for BMI at age 18-24 years
(c) Linear trend per 5 unit difference fitted across BMI values from 18.5 to 49.9 kg/m^2

© 2018 American Medical Association. All rights reserved.

eTable 3: Relative risk of premenopausal breast cancer in relation to BMI, by age at BMI and invasiveness of breast cancer

		Body-mass index category, kg/m²				Trend per 5 kg/m² BMI (b)	
		<18.5	18.5-24.9	25.0-29.9	≥ 30.0		
Age at BMI and invasiveness of breast cancer	No. of cases	HR (95% CI) (a)	HR (95% CI) (a)	HR (95% CI) (a)	HR (95% CI) (a)	HR (95% CI) (a)	P trend
BMI at age 18-24 years							
Invasive	7738	1.14 (1.07-1.21)	1.00 (ref)	0.75 (0.68-0.82)	0.55 (0.45-0.68)	0.77 (0.74-0.81)	<0.001
In-situ	1582	1.11 (0.96-1.27)	1.00 (ref)	0.71 (0.57-0.88)	0.61 (0.39-0.93)	0.71 (0.64-0.80)	<0.001
				P int=0.92		P int=0.20	
BMI at age 25-34 years							
Invasive	3109	1.25 (1.07-1.46)	1.00 (ref)	0.88 (0.79-0.97)	0.71 (0.61-0.82)	0.88 (0.84-0.92)	<0.001
In-situ	707	1.23 (0.90-1.68)	1.00 (ref)	0.59 (0.47-0.75)	0.47 (0.33-0.68)	0.76 (0.69-0.85)	<0.001
				P int=0.006		P int=0.02	
BMI at age 35-44 years							
Invasive	8486	0.99 (0.87-1.13)	1.00 (ref)	0.86 (0.82-0.91)	0.74 (0.68-0.80)	0.88 (0.86-0.90)	<0.001
In-situ	1775	1.54 (1.23-1.93)	1.00 (ref)	0.73 (0.64-0.83)	0.62 (0.51-0.74)	0.81 (0.76-0.86)	<0.001
				P int=<0.001		P int=0.01	
BMI at age 45-54 years							
Invasive	4676	1.01 (0.82-1.24)	1.00 (ref)	0.85 (0.79-0.91)	0.74 (0.68-0.82)	0.88 (0.85-0.91)	<0.001
In-situ	1010	1.35 (0.92-1.99)	1.00 (ref)	0.81 (0.69-0.94)	0.75 (0.62-0.91)	0.88 (0.82-0.94)	<0.001
				P int=0.51		P int=0.93	

Abbreviations: BMI, Body-mass Index; CI, confidence interval; HR, hazard ratio

(a) HRs adjusted for attained age, cohort, year of birth, age at menarche, age at first birth, number of births, time since last birth and family history of breast cancer
(b) Linear trend per 5 unit difference fitted across BMI values from 18.5 to 49.9 kg/m²

© 2018 American Medical Association. All rights reserved.

eTable 4: Characteristics of breast cancer cases, by most recent BMI category prior to diagnosis

	Most recent body-mass index (kg/m^2) (a)										All cases	
	<18.5		18.5-24.9		25.0-29.9		30.0-34.9		≥35			
	N or mean	%	N or mean	%	N or mean	%	N or mean	%	N or mean	%	N or mean	%
Mean interval between age at BMI and diagnosis, years	3.4		3.0		2.4		2.1		2.0		2.8	
Age at diagnosis, years												
<40	48	14.9	810	9.7	220	7.6	74	7.1	39	7.6	1191	9.1
40-44	91	28.2	1962	23.6	655	22.8	243	23.3	127	24.9	3078	23.5
45-49	146	45.2	3995	48.0	1355	47.1	472	45.3	237	46.4	6205	47.4
50-54	38	11.8	1564	18.8	646	22.5	252	24.2	108	21.1	2608	19.9
Race/ethnicity												
Caucasian	193	84.6	5350	90.0	1890	82.5	695	76.0	309	66.0	8437	85.7
Black	13	5.7	337	5.7	323	14.1	192	21.0	141	30.1	1006	10.2
Asian	18	7.9	168	2.8	39	1.7	9	1.0	1	0.2	235	2.4
Other	4	1.8	88	1.5	39	1.7	19	2.1	17	3.6	167	1.7
Not known	95		2388		585		126		43		3237	
Ever had mammography for screening (b)												
No	63	28.0	1442	25.7	379	20.3	119	18.1	57	16.4	2060	23.7
Yes	162	72.0	4169	74.3	1489	79.7	540	81.9	290	83.6	6650	76.3
Not known	98		2720		1008		382		164		4372	
Invasiveness												
In situ	70	21.7	1385	16.7	440	15.5	156	15.2	87	17.5	2138	16.5
Invasive	252	78.3	6904	83.3	2401	84.5	868	84.8	411	82.5	10836	83.5
Not known	1		42		35		17		13		108	
ER status												
Positive	115	71.4	3532	75.2	1352	74.0	537	73.2	263	72.9	5799	74.6
Negative	46	28.6	1163	24.8	475	26.0	197	26.8	98	27.1	1979	25.4
Borderline/not known	162		3636		1049		307		150		5304	
Combined ER and PR status												
ER+PR+	95	63.3	2744	63.2	1086	63.9	437	63.6	208	61.7	4570	63.3
ER+PR-	11	7.3	487	11.2	162	9.5	60	8.7	36	10.7	756	10.5
ER-PR+	39	26.0	836	19.3	365	21.5	158	23.0	86	25.5	1484	20.6
ER-PR-	5	3.3	274	6.3	86	5.1	32	4.7	7	2.1	404	5.6
Unclassified	173		3990		1177		354		174		5868	

© 2018 American Medical Association. All rights reserved.

eTable 4:continued

	Most recent body-mass index (kg/m²) (a)										All cases	
	<18.5		18.5-24.9		25.0-29.9		30.0-34.9		≥35			
	N or mean	%	N or mean	%	N or mean	%	N or mean	%	N or mean	%	N or mean	%
Clinicopathological surrogate definition of intrinsic tumour subtype (c)												
Luminal A-like	40	25.0	1201	25.8	514	28.4	206	28.2	97	27.2	2058	26.7
Luminal B-like HER2-	8	5.0	240	5.2	82	4.5	35	4.8	17	4.8	382	5.0
Luminal B-like HER2+	14	8.8	287	6.2	131	7.2	62	8.5	22	6.2	516	6.7
Luminal B-like, unclassified	7	4.4	444	9.5	135	7.5	41	5.6	21	5.9	648	8.4
HER2-enriched	3	1.9	121	2.6	54	3.0	19	2.6	10	2.8	207	2.7
Triple-negative	10	6.3	269	5.8	116	6.4	53	7.3	35	9.8	483	6.3
Unclassified luminal	52	32.5	1644	35.3	584	32.2	228	31.2	113	31.7	2621	34.0
Unclassified non-luminal	26	16.3	446	9.6	195	10.8	86	11.8	41	11.5	794	10.3
Unclassified	163		3679		1065		311		155		5373	
All cases of breast cancer	323	100.0	8331	100.0	2876	100.0	1041	100.0	511	100.0	13082	100.0

Abbreviations: ER, oestrogen-receptor; HER2, Human Epidermal Growth Factor Receptor-2; PR, progesterone-receptor

(a) Most recently assessed BMI prior to diagnosis
(b) Most recent mammographic screening status prior to diagnosis
(c) Luminal A-like: ER+PR+HER2-
Luminal B-like, all: All ER+ and/or PR+ tumours that are not ER+PR+HER2- with subtypes Luminal B-like, HER2-: ER+PR−HER2- and ER−PR+HER2- and Luminal B-like, HER2+: [ER+ and/or PR+] and HER2+
Non-luminal, all: ER−PR− regardless of HER2 status with subtypes HER2-enriched: ER−PR−HER2+ and Triple-negative: ER−PR−HER2-

© 2018 American Medical Association. All rights reserved.

eTable 5: Relative risk of premenopausal breast cancer in relation to BMI category, by age at BMI and oestrogen or progesterone receptor status of breast cancer

		Body-mass index category, kg/m²					
		<18.5	18.5-24.9	25.0-29.9	≥30.0	Trend per 5 kg/m² (b)	
**Age at BMI and hormone-receptor status**	**No. of cases**	**HR (95% CI) (a)**	**HR (95% CI) (a)**	**HR (95% CI) (a)**	**HR (95% CI) (a)**	**HR (95% CI) (a)**	**P trend**
BMI age 18-24 years							
Oestrogen-receptor							
ER+	4436	1.15 (1.06-1.25)	1.00 (ref)	0.75 (0.66-0.84)	0.55 (0.43-0.72)	0.76 (0.71-0.81)	<0.001
ER-	1478	1.18 (1.02-1.35)	1.00 (ref)	0.79 (0.65-0.97)	0.45 (0.28-0.72)	0.81 (0.74-0.90)	<0.001
Progesterone-receptor				P int=0.82(c)		P int=0.25(c)	
PR+	3710	1.12 (1.03-1.23)	1.00 (ref)	0.74 (0.64-0.84)	0.47 (0.35-0.63)	0.74 (0.70-0.80)	<0.001
PR-	1723	1.20 (1.05-1.37)	1.00 (ref)	0.77 (0.63-0.93)	0.58 (0.40-0.86)	0.80 (0.73-0.88)	<0.001
BMI age 25-34 years				P int=0.70(c)		P int=0.21(c)	
Oestrogen-receptor							
ER+	1884	1.37 (1.13-1.65)	1.00 (ref)	0.81 (0.71-0.92)	0.64 (0.53-0.77)	0.85 (0.80-0.90)	<0.001
ER-	613	1.03 (0.70-1.52)	1.00 (ref)	0.89 (0.72-1.10)	0.80 (0.60-1.06)	0.90 (0.83-0.99)	0.025
Progesterone-receptor				P int=0.24(c)		P int=0.26(c)	
PR+	1626	1.30 (1.05-1.61)	1.00 (ref)	0.83 (0.72-0.95)	0.58 (0.47-0.71)	0.83 (0.78-0.88)	<0.001
PR-	719	1.25 (0.90-1.75)	1.00 (ref)	0.86 (0.70-1.05)	0.90 (0.70-1.16)	0.94 (0.86-1.02)	0.122
BMI age 35-44 years				P int=0.06(c)		P int=0.02(c)	
Oestrogen-receptor							
ER+	4904	1.02 (0.86-1.21)	1.00 (ref)	0.83 (0.77-0.90)	0.71 (0.64-0.78)	0.87 (0.84-0.90)	<0.001
ER-	1588	1.06 (0.78-1.43)	1.00 (ref)	0.96 (0.85-1.09)	0.81 (0.68-0.95)	0.92 (0.87-0.97)	0.001
Progesterone-receptor				P int=0.20(c)		P int=0.07(c)	
PR+	4213	1.04 (0.87-1.25)	1.00 (ref)	0.84 (0.77-0.91)	0.71 (0.64-0.79)	0.86 (0.83-0.89)	<0.001
PR-	1830	1.05 (0.80-1.39)	1.00 (ref)	0.91 (0.81-1.02)	0.79 (0.68-0.92)	0.91 (0.86-0.95)	<0.001
BMI age 45-54 years				P int=0.52(c)		P int=0.08(c)	

© 2018 American Medical Association. All rights reserved.

Oestrogen-receptor							
ER+	3016	0.76 (0.56-1.02)	1.00 (ref)	0.86 (0.79-0.94)	0.77 (0.69-0.86)	0.90 (0.86-0.93)	<0.001
ER-	867	1.28 (0.83-1.98)	1.00 (ref)	0.81 (0.69-0.96)	0.75 (0.61-0.91)	0.90 (0.84-0.97)	0.004
				P int=0.22(c)		P int=0.84(c)	
Progesterone-receptor							
PR+	2589	0.83 (0.61-1.13)	1.00 (ref)	0.86 (0.78-0.95)	0.79 (0.70-0.89)	0.91 (0.87-0.95)	<0.001
PR-	1064	1.07 (0.70-1.63)	1.00 (ref)	0.80 (0.69-0.93)	0.71 (0.59-0.86)	0.87 (0.82-0.93)	<0.001
				P int=0.49(c)		P int=0.26(c)	

Abbreviations: BMI, Body-mass Index; CI, confidence interval; ER, oestrogen-receptor; HR, hazard ratio; PR, progesterone-receptor

(a) HRs adjusted for attained age, cohort, year of birth, age at menarche, age at first birth, number of births, time since last birth and family history of breast cancer

(b) Linear trend per 5 unit difference fitted across BMI values from 18.5 to 49.9 kg/m^2

(c) Tests for heterogeneity in effect by ER or PR status obtained from an Augmentation model.[8]

© 2018 American Medical Association. All rights reserved.

eTable 6: Relative risk of premenopausal breast cancer per 5 kg/m^2 difference in BMI, by age at BMI and other breast cancer risk factors

	Age at BMI, years							
	BMI at age 18-24 years		BMI at age 25-34 years		BMI at age 35-44 years		BMI at age 45-54 years	
Variable (a)	No. of cases	HR (95% CI) (b)	No. of cases	HR (95% CI) (b)	No. of cases	HR (95% CI) (b)	No. of cases	HR (95% CI) (b)
Adult height, cms								
<160	1378	0.79 (0.71-0.87)	666	0.86 (0.78-0.95)	1881	0.88 (0.84-0.93)	1101	0.88 (0.82-0.94)
160-169	4378	0.76 (0.71-0.80)	1901	0.85 (0.81-0.90)	5509	0.86 (0.83-0.89)	3156	0.87 (0.84-0.90)
≥170	2152	0.79 (0.72-0.85)	1087	0.85 (0.79-0.92)	2641	0.89 (0.85-0.93)	1349	0.92 (0.87-0.97)
		P int=0.65		P int=0.98		P int=0.46		P int=0.26
Age at menarche, years								
<12	1806	0.78 (0.72-0.84)	846	0.90 (0.84-0.97)	2187	0.88 (0.85-0.92)	1209	0.92 (0.87-0.97)
12-13	4386	0.77 (0.72-0.81)	2067	0.83 (0.78-0.88)	5461	0.87 (0.84-0.90)	3098	0.87 (0.83-0.90)
≥14	1562	0.75 (0.67-0.84)	671	0.84 (0.75-0.94)	2111	0.84 (0.79-0.89)	1180	0.89 (0.83-0.96)
		P int=0.89		P int=0.17		P int=0.41		P int=0.20
Ever having had a birth								
No	1200	0.72 (0.66-0.79)	638	0.79 (0.73-0.87)	1436	0.84 (0.80-0.89)	698	0.85 (0.79-0.91)
Yes	6461	0.78 (0.74-0.82)	2823	0.88 (0.84-0.93)	8180	0.88 (0.85-0.90)	4734	0.89 (0.86-0.92)
		P int=0.16		P int=0.03		P int=0.15		P int=0.18
Age at first birth, years (parous)								
<25	2472	0.77 (0.71-0.83)	885	0.89 (0.82-0.96)	3285	0.86 (0.82-0.90)	2040	0.90 (0.86-0.94)
25-34	3550	0.78 (0.73-0.83)	1758	0.87 (0.82-0.93)	4333	0.88 (0.85-0.92)	2396	0.88 (0.84-0.92)
≥35	383	0.81 (0.67-0.97)	161	0.84 (0.68-1.03)	522	0.91 (0.82-1.00)	283	0.93 (0.83-1.05)
		P int=0.89		P int=0.84		P int=0.45		P int=0.63
Number of births (parous)								
1	1160	0.82 (0.74-0.90)	603	0.82 (0.74-0.91)	1504	0.87 (0.82-0.92)	787	0.92 (0.86-0.99)
2	3061	0.79 (0.74-0.86)	1355	0.90 (0.84-0.97)	3893	0.89 (0.85-0.92)	2152	0.91 (0.87-0.96)
≥3	2191	0.72 (0.65-0.79)	816	0.86 (0.78-0.95)	2676	0.86 (0.82-0.90)	1763	0.85 (0.81-0.90)
		P int=0.12		P int=0.26		P int=0.63		P int=0.09
Time since last birth, years (parous)								
<5	298	0.89 (0.72-1.09)	269	0.89 (0.77-1.04)	281	0.86 (0.75-0.99)	21	1.19 (0.81-1.74)
5-9	814	0.84 (0.74-0.96)	486	0.86 (0.76-0.96)	1036	0.90 (0.83-0.96)	224	0.88 (0.76-1.02)
10-14	1431	0.75 (0.68-0.84)	719	0.86 (0.78-0.95)	1868	0.88 (0.84-0.93)	729	0.87 (0.80-0.94)

© 2018 American Medical Association. All rights reserved.

eTable 6: continued

Variable (a)	Age at BMI, years							
	BMI at age 18-24 years		BMI at age 25-34 years		BMI at age 35-44 years		BMI at age 45-54 years	
	No. of cases	HR (95% CI) (b)	No. of cases	HR (95% CI) (b)	No. of cases	HR (95% CI) (b)	No. of cases	HR (95% CI) (b)
15-19	1715	0.76 (0.69-0.84)	662	0.87 (0.79-0.96)	2307	0.87 (0.83-0.92)	1446	0.89 (0.84-0.94)
≥20	1691	0.77 (0.70-0.85)	499	0.91 (0.81-1.01)	2231	0.86 (0.81-0.90)	2131	0.90 (0.86-0.95)
		P int=0.66		P int=0.99		P int=0.85		P int=0.43
Ever used oral contraception								
No	1267	0.68 (0.61-0.76)	384	0.85 (0.75-0.97)	2026	0.81 (0.77-0.86)	2044	0.85 (0.81-0.90)
Yes	6045	0.79 (0.75-0.83)	2979	0.86 (0.82-0.90)	7161	0.88 (0.86-0.91)	3319	0.90 (0.87-0.94)
		P int=0.02		P int=0.84		P int=0.009		P int=0.08
Ever diagnosis with infertility								
No	3525	0.78 (0.73-0.83)	1767	0.84 (0.79-0.90)	4220	0.89 (0.86-0.92)	1752	0.92 (0.88-0.96)
Yes	813	0.74 (0.65-0.85)	415	0.86 (0.76-0.96)	1089	0.85 (0.80-0.91)	573	0.90 (0.83-0.98)
		P int=0.52		P int=0.82		P int=0.30		P int=0.63
Ever diagnosis with PCOS								
No	1015	0.78 (0.69-0.90)	417	0.81 (0.71-0.94)	1253	0.88 (0.82-0.94)	561	0.92 (0.84-1.01)
Yes	217	0.67 (0.53-0.86)	110	0.72 (0.58-0.91)	262	0.78 (0.69-0.89)	143	0.84 (0.73-0.98)
		P int=0.28		P int=0.39		P int=0.12		P int=0.30
Ever had a mammogram								

© 2018 American Medical Association. All rights reserved.

No	1322	0.84 (0.75-0.93)	647	0.91 (0.83-1.00)	1389	0.89 (0.83-0.95)	421	0.91 (0.81-1.02)
Yes	3706	0.76 (0.71-0.81)	1378	0.83 (0.77-0.88)	5393	0.87 (0.84-0.89)	3549	0.88 (0.85-0.92)
		P int=0.12		P int=0.08		P int=0.52		P int=0.67
Family history of breast cancer								
No	5889	0.75 (0.72-0.79)	2749	0.86 (0.82-0.90)	6800	0.87 (0.84-0.89)	3814	0.88 (0.85-0.91)
Yes	1610	0.80 (0.73-0.87)	768	0.83 (0.75-0.91)	2312	0.89 (0.85-0.93)	1446	0.90 (0.86-0.95)
		P int=0.32		P int=0.45		P int=0.34		P int=0.41
Attained education level								
Primary and lower	325	0.84 (0.66-1.05)	35	0.90 (0.61-1.32)	318	0.76 (0.64-0.89)	226	0.82 (0.68-0.99)
High School	485	0.80 (0.67-0.96)	204	0.84 (0.72-0.99)	522	0.88 (0.80-0.97)	203	0.84 (0.72-0.98)
Some college education	1320	0.81 (0.73-0.90)	831	0.89 (0.82-0.96)	1687	0.86 (0.82-0.91)	563	0.90 (0.83-0.98)
University	4821	0.75 (0.71-0.79)	2288	0.85 (0.80-0.90)	5502	0.88 (0.85-0.91)	3343	0.88 (0.85-0.92)
		P int=0.51		P int=0.82		P int=0.30		P int=0.72

eTable 6: continued

	Age at BMI, years							
	BMI at age 18-24 years		BMI at age 25-34 years		BMI at age 35-44 years		BMI at age 45-54 years	
Variable (a)	No. of cases	HR (95% CI) (b)	No. of cases	HR (95% CI) (b)	No. of cases	HR (95% CI) (b)	No. of cases	HR (95% CI) (b)
Ethnicity								
Caucasian	5922	0.73 (0.70-0.77)	2836	0.83 (0.79-0.88)	6708	0.86 (0.84-0.89)	3780	0.88 (0.85-0.91)
Black	709	0.84 (0.76-0.93)	460	0.90 (0.83-0.98)	820	0.90 (0.85-0.95)	282	0.91 (0.83-1.01)
Asian	79	0.69 (0.40-1.18)	93	0.89 (0.61-1.31)	124	1.02 (0.77-1.36)	90	0.80 (0.57-1.12)
		P int=0.08		P int=0.29		P int=0.30		P int=0.68
Continent								
North-America	5603	0.76 (0.72-0.80)	2960	0.86 (0.82-0.90)	6669	0.88 (0.86-0.90)	3658	0.89 (0.86-0.92)

© 2018 American Medical Association. All rights reserved.

Europe	2236	0.80 (0.73-0.88)	657	0.81 (0.72-0.90)	3279	0.84 (0.80-0.89)	1857	0.85 (0.79-0.91)
Asia			37	1.79 (0.95-3.40)	38	0.75 (0.39-1.44)	37	0.71 (0.39-1.28)
Australia	69	0.65 (0.38-1.12)	2960	0.86 (0.82-0.90)	45	0.68 (0.45-1.02)	54	1.06 (0.79-1.40)
		P int=0.48		P int=0.07		P int=0.25		P int=0.33
Year of birth								
<1940	976	0.75 (0.66-0.85)	22	1.55 (0.66-3.66)	1012	0.85 (0.78-0.92)	1083	0.84 (0.78-0.91)
1940-1949	1770	0.72 (0.65-0.79)	777	0.83 (0.74-0.92)	2912	0.83 (0.79-0.88)	2410	0.86 (0.82-0.90)
1950-1959	3623	0.78 (0.73-0.83)	1461	0.86 (0.80-0.92)	4563	0.88 (0.85-0.91)	1703	0.90 (0.86-0.95)
≥1960	1539	0.80 (0.73-0.87)	1394	0.85 (0.80-0.91)	1544	0.90 (0.85-0.94)	410	0.94 (0.87-1.03)
		P int=0.41		P int=0.58		P int=0.20		P int=0.11

Abbreviations: BMI, Body-mass Index; CI, confidence interval; HR, hazard ratio; PCOS, polycystic ovary syndrome

(a) Stratifying variables are time-updated, where possible, for reproductive variables, oral contraceptive use, infertility, PCOS, screening mammogram and family history of breast cancer.

(b) Hazard ratio represents linear trend per 5 kg/m^2 difference fitted across BMI values from 18.5 to 49.9 kg/m^2 and are adjusted for attained age, cohort, year of birth, age at menarche, age at first birth, number of births, time since last birth and family history of breast cancer.

© 2018 American Medical Association. All rights reserved.

eTable 7: Relative risk of premenopausal breast cancer in relation to BMI category, by age at BMI. For (1) all subjects included in main analysis (2) breast cancer with known ER, PR and HER2 status as endpoint (3) excluding subjects with weight assessed less than three years postpartum (4) strictly known premenopausal time only (5) excluding first two years of follow-up.

	All subjects		Cases with information on all of ER, PR and HER2 status only		Excluding subjects with weights recalled or reported <3 years postpartum		Analyses restricted to strictly known premenopausal time only		Analyses excluding first two years of follow-up	
**Age at BMI and BMI category, kg/m²**	**No. of cases**	**HR (95% CI) (a)**	**No. of cases**	**HR (95% CI) (a)**	**No. of cases**	**HR (95% CI) (a)**	**No. of cases**	**HR (95% CI) (a)**	**No. of cases**	**HR (95% CI) (a)**
BMI at age 18-24 years										
15.0-16.9	328	1.15 (1.03-1.29)	87	1.09 (0.88-1.35)	293	1.12 (1.00-1.26)	184	1.20 (1.04-1.40)	283	1.15 (1.02-1.30)
17.0-18.4	1169	1.09 (1.02-1.16)	342	1.08 (0.96-1.21)	1068	1.08 (1.01-1.15)	636	1.08 (1.00-1.18)	998	1.07 (1.00-1.15)
18.5-22.9	6364	1.00 (ref)	2046	1.00 (ref)	5924	1.00 (ref)	3701	1.00 (ref)	5529	1.00 (ref)
23.0-24.9	864	0.80 (0.75-0.86)	277	0.78 (0.69-0.88)	793	0.80 (0.74-0.86)	470	0.77 (0.70-0.85)	758	0.81 (0.75-0.88)
25.0-27.4	418	0.73 (0.66-0.81)	137	0.71 (0.60-0.84)	384	0.73 (0.66-0.81)	251	0.75 (0.66-0.85)	367	0.73 (0.66-0.81)
27.5-29.9	141	0.68 (0.58-0.81)	54	0.76 (0.58-0.99)	127	0.67 (0.56-0.80)	78	0.63 (0.51-0.79)	128	0.70 (0.58-0.83)
30.0-32.4	81	0.73 (0.58-0.90)	21	0.55 (0.36-0.84)	72	0.70 (0.56-0.89)	44	0.67 (0.50-0.90)	74	0.74 (0.59-0.93)
32.5-34.9	25	0.47 (0.32-0.70)	7	0.40 (0.19-0.84)	23	0.48 (0.32-0.72)	11	0.39 (0.22-0.71)	19	0.40 (0.25-0.62)
35.0-49.9	15	0.27 (0.16-0.45)	6	0.35 (0.16-0.77)	15	0.30 (0.18-0.50)	7	0.26 (0.12-0.55)	13	0.26 (0.15-0.45)
Trend per 5 kg/m² (b)	7908	0.77 (0.73-0.80)	2548	0.75 (0.69-0.81)	7338	0.76 (0.73-0.80)	4562	0.74 (0.70-0.78)	6888	0.77 (0.73-0.80)
BMI at age 25-34 years										
15.0-18.4	221	1.22 (1.06-1.40)	85	1.25 (1.00-1.56)	151	1.29 (1.09-1.53)	132	1.22 (1.02-1.46)	203	1.20 (1.04-1.38)
18.5-22.9	2222	1.00 (ref)	834	1.00 (ref)	1405	1.00 (ref)	1359	1.00 (ref)	2130	1.00 (ref)

© 2018 American Medical Association. All rights reserved.

23.0-24.9	607	0.91 (0.83-0.99)	213	0.87 (0.74-1.01)	412	0.99 (0.89-1.11)	347	0.92 (0.82-1.03)	575	0.90 (0.82-0.99)
25.0-27.4	403	0.82 (0.74-0.91)	138	0.79 (0.66-0.95)	259	0.84 (0.74-0.97)	216	0.82 (0.71-0.95)	390	0.83 (0.74-0.92)
27.5-29.9	176	0.76 (0.65-0.88)	66	0.83 (0.64-1.06)	127	0.86 (0.71-1.03)	97	0.85 (0.69-1.05)	167	0.75 (0.64-0.88)
30.0-32.4	101	0.67 (0.54-0.81)	34	0.66 (0.47-0.93)	71	0.70 (0.55-0.88)	49	0.64 (0.48-0.85)	99	0.68 (0.56-0.84)
32.5-34.9	58	0.63 (0.49-0.82)	21	0.70 (0.45-1.08)	39	0.61 (0.44-0.84)	28	0.65 (0.45-0.95)	56	0.64 (0.49-0.83)
35.0-37.4	38	0.67 (0.48-0.92)	11	0.59 (0.33-1.08)	29	0.72 (0.50-1.05)	17	0.63 (0.39-1.01)	37	0.68 (0.49-0.94)
37.5-39.9	24	0.64 (0.42-0.95)	4	0.35 (0.13-0.95)	13	0.49 (0.28-0.84)	6	0.38 (0.17-0.85)	23	0.63 (0.42-0.96)
40.0-49.9	25	0.49 (0.33-0.74)	9	0.66 (0.34-1.27)	17	0.43 (0.27-0.70)	9	0.46 (0.24-0.89)	24	0.49 (0.33-0.74)

eTable 7: continued

	All subjects		Cases with information on all of ER, PR and HER2 status only		Excluding subjects with weights recalled or reported <3 years postpartum		Analyses restricted to strictly known premenopausal time only		Analyses excluding first two years of follow-up	
**Age at BMI and BMI category, kg/m²**	**No. of cases**	**HR (95% CI) (a)**	**No. of cases**	**HR (95% CI) (a)**	**No. of cases**	**HR (95% CI) (a)**	**No. of cases**	**HR (95% CI) (a)**	**No. of cases**	**HR (95% CI) (a)**
Trend per 5 kg/m² (b)	3654	0.85 (0.82-0.89)	1330	0.85 (0.80-0.92)	2372	0.85 (0.81-0.90)	2128	0.85 (0.80-0.90)	3501	0.85 (0.82-0.89)
BMI at age 35-44 years										
15.0-18.4	318	1.05 (0.94-1.18)	91	1.14 (0.92-1.40)	256	1.06 (0.93-1.20)	234	1.13 (0.99-1.29)	283	1.06 (0.94-1.20)
18.5-22.9	5340	1.00 (ref)	1556	1.00 (ref)	4441	1.00 (ref)	3595	1.00 (ref)	4724	1.00 (ref)
23.0-24.9	1882	0.91 (0.86-0.96)	576	0.93 (0.85-1.03)	1583	0.91 (0.86-0.97)	1129	0.90 (0.84-0.96)	1683	0.93 (0.88-0.98)
25.0-27.4	1264	0.82 (0.77-0.88)	389	0.84 (0.75-0.94)	1073	0.83 (0.77-0.89)	741	0.84 (0.78-0.91)	1127	0.83 (0.78-0.89)
27.5-29.9	636	0.81 (0.74-0.88)	211	0.86 (0.74-0.99)	563	0.84 (0.77-0.92)	358	0.82 (0.73-0.91)	551	0.79 (0.72-0.87)

© 2018 American Medical Association. All rights reserved.

30.0-32.4	417	0.81 (0.73-0.90)	135	0.85 (0.71-1.01)	364	0.83 (0.75-0.93)	225	0.81 (0.70-0.92)	375	0.82 (0.74-0.91)
32.5-34.9	214	0.70 (0.61-0.80)	78	0.81 (0.64-1.02)	186	0.70 (0.60-0.81)	110	0.66 (0.55-0.80)	188	0.69 (0.59-0.79)
35.0-37.4	123	0.63 (0.53-0.75)	41	0.65 (0.48-0.89)	106	0.62 (0.51-0.75)	61	0.57 (0.45-0.74)	110	0.63 (0.52-0.76)
37.5-39.9	74	0.60 (0.48-0.76)	20	0.53 (0.34-0.82)	63	0.59 (0.46-0.76)	31	0.49 (0.34-0.70)	61	0.55 (0.43-0.71)
40.0-49.9	81	0.49 (0.39-0.61)	20	0.39 (0.25-0.60)	73	0.50 (0.39-0.63)	36	0.43 (0.31-0.59)	79	0.52 (0.42-0.66)
Trend per 5 kg/m^2 (b)	1003 1	0.87 (0.85-0.89)	3026	0.88 (0.85-0.92)	8452	0.87 (0.85-0.89)	6286	0.86 (0.83-0.89)	8898	0.87 (0.85-0.89)
BMI at age 45-54 years										
15.0-18.4	120	1.04 (0.87-1.25)	23	0.92 (0.60-1.39)	120	1.08 (0.90-1.30)	102	1.06 (0.87-1.29)	103	1.10 (0.90-1.34)
18.5-22.9	2484	1.00 (ref)	668	1.00 (ref)	2436	1.00 (ref)	2063	1.00 (ref)	2037	1.00 (ref)
23.0-24.9	1127	0.94 (0.87-1.01)	366	1.01 (0.89-1.14)	1104	0.93 (0.87-1.00)	869	0.91 (0.84-0.99)	933	0.95 (0.88-1.03)
25.0-27.4	859	0.85 (0.79-0.92)	303	0.91 (0.79-1.04)	844	0.85 (0.79-0.92)	649	0.84 (0.77-0.92)	710	0.86 (0.78-0.93)
27.5-29.9	430	0.77 (0.69-0.85)	176	0.89 (0.76-1.06)	429	0.78 (0.70-0.86)	331	0.78 (0.70-0.88)	348	0.75 (0.67-0.84)
30.0-32.4	302	0.80 (0.71-0.90)	117	0.84 (0.69-1.02)	302	0.81 (0.71-0.91)	221	0.77 (0.67-0.89)	260	0.81 (0.71-0.92)
32.5-34.9	160	0.72 (0.61-0.84)	68	0.79 (0.61-1.02)	159	0.72 (0.61-0.85)	113	0.68 (0.56-0.82)	136	0.70 (0.59-0.84)
35.0-37.4	112	0.76 (0.63-0.92)	58	0.98 (0.74-1.28)	112	0.76 (0.63-0.93)	86	0.77 (0.62-0.96)	96	0.74 (0.60-0.91)
37.5-39.9	62	0.66 (0.52-0.86)	21	0.56 (0.36-0.86)	62	0.67 (0.52-0.87)	42	0.60 (0.44-0.82)	57	0.69 (0.53-0.89)
40.0-49.9	70	0.56 (0.44-0.71)	33	0.65 (0.46-0.92)	69	0.55 (0.43-0.71)	48	0.52 (0.39-0.69)	61	0.54 (0.42-0.70)

eTable 7: continued

	All subjects		Cases with information on all of ER, PR and HER2 status only		Excluding subjects with weights recalled or reported <3 years postpartum		Analyses restricted to strictly known premenopausal time only		Analyses excluding first two years of follow-up	
**Age at BMI and BMI**	**No. of**	**HR (95% CI) (a)**	**No. of**	**HR (95% CI) (a)**	**No. of**	**HR (95% CI) (a)**	**No. of**	**HR (95% CI) (a)**	**No. of cases**	**HR (95% CI) (a)**

© 2018 American Medical Association. All rights reserved.

category, kg/m^2	cases		cases		cases		cases			
Trend per 5 kg/m^2 (b)	5606	0.88 (0.86-0.91)	1810	0.92 (0.87-0.96)	5517	0.88 (0.86-0.91)	4422	0.88 (0.85-0.91)	4638	0.88 (0.85-0.91)

Abbreviations: BMI, Body-mass Index; HR, hazard ratio; CI, confidence interval; HER2, human epidermal growth factor receptor-2; ER, oestrogen-receptor; PR, progesterone-receptor

(a) HRs adjusted for attained age, cohort, year of birth, age at menarche, age at first birth, number of births, time since last birth and family history of breast cancer
(b) Linear trend per 5 unit difference fitted across BMI values from 18.5 to 49.9 kg/m^2

© 2018 American Medical Association. All rights reserved.

eTable 8: Relative risk of premenopausal breast cancer in relation to BMI category at age 18-24 years, excluding subjects contributing to each successive cohort.

		Body-mass index category, kg/m^2						
		<18.5	18.5-24.9	25.0-29.9	30.0-34.9	≥ 35.0	Trend per 5 kg/m^2 (b)	
Excluded cohort	No. of cases	HR (95% CI)(a)	HR (95% CI)	HR (95% CI)(a)	HR (95% CI)(a)	HR (95% CI)(a)	HR (95% CI)(a)	P trend
No exclusions	9405	1.13 (1.07-1.20)	1.00 (ref)	0.74 (0.68-0.81)	0.66 (0.55-0.81)	0.28 (0.17-0.47)	0.77 (0.73-0.80)	<0.001
BWHS	8585	1.11 (1.05-1.18)	1.00 (ref)	0.76 (0.69-0.83)	0.64 (0.51-0.79)	0.23 (0.12-0.44)	0.75 (0.72-0.79)	<0.001
CLUE2	9341	1.13 (1.07-1.20)	1.00 (ref)	0.74 (0.68-0.81)	0.67 (0.56-0.82)	0.29 (0.17-0.47)	0.77 (0.73-0.80)	<0.001
CSDLH	9192	1.14 (1.07-1.20)	1.00 (ref)	0.74 (0.68-0.81)	0.66 (0.54-0.80)	0.28 (0.17-0.47)	0.77 (0.73-0.80)	<0.001
CTS	8769	1.13 (1.07-1.20)	1.00 (ref)	0.74 (0.68-0.81)	0.67 (0.55-0.82)	0.30 (0.17-0.50)	0.77 (0.74-0.81)	<0.001
E3N (c)	-	-	-	-	-	-	-	-
EPIC	8993	1.14 (1.08-1.21)	1.00 (ref)	0.74 (0.68-0.81)	0.66 (0.54-0.80)	0.29 (0.17-0.48)	0.76 (0.73-0.80)	<0.001
GS	8764	1.13 (1.07-1.20)	1.00 (ref)	0.73 (0.67-0.80)	0.65 (0.53-0.80)	0.30 (0.18-0.49)	0.76 (0.73-0.80)	<0.001
HUNT2 (d)	-	-	-	-	-	-	-	-
MCC	9322	1.13 (1.07-1.20)	1.00 (ref)	0.74 (0.68-0.81)	0.67 (0.55-0.81)	0.28 (0.17-0.47)	0.77 (0.73-0.80)	<0.001
NHS	7428	1.12 (1.05-1.19)	1.00 (ref)	0.76 (0.69-0.84)	0.65 (0.53-0.81)	0.26 (0.15-0.45)	0.79 (0.75-0.83)	<0.001
NHS2	6912	1.14 (1.07-1.22)	1.00 (ref)	0.72 (0.65-0.80)	0.71 (0.57-0.89)	0.30 (0.17-0.55)	0.76 (0.72-0.80)	<0.001
NOWAC	8638	1.14 (1.08-1.21)	1.00 (ref)	0.73 (0.67-0.80)	0.65 (0.53-0.79)	0.29 (0.17-0.48)	0.76 (0.73-0.80)	<0.001
NYUWHS (c)	-	-	-	-	-	-	-	-
RERF (d)	-	-	-	-	-	-	-	-
SCHS (c)	-	-	-	-	-	-	-	-
SIS (c)	-	-	-	-	-	-	-	-
SMC	9195	1.14 (1.07-1.20)	1.00 (ref)	0.74 (0.68-0.81)	0.66 (0.55-0.80)	0.28 (0.17-0.47)	0.76 (0.73-0.80)	<0.001
USRTC	8948	1.13 (1.07-1.20)	1.00 (ref)	0.74 (0.68-0.81)	0.69 (0.56-0.83)	0.28 (0.16-0.47)	0.77 (0.73-0.80)	<0.001
WLHS	8773	1.13 (1.07-1.20)	1.00 (ref)	0.74 (0.68-0.81)	0.67 (0.56-0.82)	0.29 (0.17-0.48)	0.77 (0.73-0.80)	<0.001

Abbreviations: BMI, Body-mass Index; CI, confidence interval; HR, hazard ratio; for cohort abbreviations see page 3.
(a) HRs adjusted for attained age, cohort, year of birth, age at menarche, age at first birth, number of births, time since last birth and family history of breast cancer
(b) Linear trend per 5 unit difference fitted across BMI values from 18.5 to 49.9 kg/m^2
(c) BMI data at age 18-24 years not collected for these cohorts
(d) Insufficient number of cases with BMI at age 18-24 years for these cohorts

© 2018 American Medical Association. All rights reserved.

eTable 9: Relative risk of premenopausal breast cancer in relation to BMI category at age 45-54 years, excluding subjects contributing to each successive cohort.

Abbreviations: BMI, Body-mass Index; CI, confidence interval; HR, hazard ratio; for cohort abbreviations see page 3.

		Body-mass index category, kg/m^2						
		<18.5	18.5-24.9	25.0-29.9	30.0-34.9	≥ 35.0	Trend per 5 kg/m^2 (b)	
**Excluded cohort**	**No. of cases**	**HR (95% CI) (a)**	**HR (95% CI)**	**HR (95% CI) (a)**	**HR (95% CI) (a)**	**HR (95% CI) (a)**	**HR (95% CI)(a)**	**P trend**
No exclusions	5726	1.06 (0.88-1.27)	1.00 (ref)	0.84 (0.79-0.90)	0.79 (0.71-0.87)	0.68 (0.60-0.78)	0.88 (0.86-0.91)	<0.001
BWHS	5515	1.06 (0.89-1.28)	1.00 (ref)	0.85 (0.80-0.91)	0.78 (0.70-0.87)	0.69 (0.60-0.80)	0.88 (0.86-0.91)	<0.001
CLUE2	5704	1.06 (0.89-1.28)	1.00 (ref)	0.84 (0.79-0.90)	0.78 (0.71-0.86)	0.69 (0.60-0.79)	0.88 (0.86-0.91)	<0.001
CSDLH	5707	1.06 (0.88-1.27)	1.00 (ref)	0.85 (0.79-0.90)	0.78 (0.71-0.87)	0.68 (0.60-0.78)	0.88 (0.86-0.91)	<0.001
CTS	5593	1.07 (0.89-1.29)	1.00 (ref)	0.84 (0.79-0.90)	0.78 (0.70-0.86)	0.68 (0.60-0.78)	0.88 (0.85-0.91)	<0.001
E3N	4719	1.03 (0.82-1.30)	1.00 (ref)	0.84 (0.78-0.90)	0.79 (0.71-0.87)	0.67 (0.59-0.77)	0.88 (0.85-0.91)	<0.001
EPIC	5596	1.08 (0.90-1.29)	1.00 (ref)	0.84 (0.79-0.90)	0.79 (0.71-0.87)	0.68 (0.59-0.78)	0.89 (0.86-0.91)	<0.001
GS	5481	1.06 (0.89-1.28)	1.00 (ref)	0.84 (0.78-0.90)	0.78 (0.71-0.87)	0.68 (0.59-0.77)	0.88 (0.85-0.91)	<0.001
HUNT2	5707	1.06 (0.89-1.28)	1.00 (ref)	0.85 (0.79-0.90)	0.78 (0.71-0.87)	0.68 (0.60-0.78)	0.88 (0.86-0.91)	<0.001
MCC	5672	1.07 (0.89-1.28)	1.00 (ref)	0.84 (0.79-0.90)	0.78 (0.71-0.87)	0.68 (0.59-0.77)	0.88 (0.85-0.91)	<0.001
NHS	4159	1.04 (0.85-1.29)	1.00 (ref)	0.86 (0.80-0.93)	0.83 (0.74-0.93)	0.71 (0.61-0.83)	0.90 (0.87-0.93)	<0.001
NHS2	4431	1.02 (0.84-1.25)	1.00 (ref)	0.82 (0.77-0.89)	0.78 (0.69-0.88)	0.66 (0.56-0.78)	0.87 (0.84-0.90)	<0.001
NOWAC	5494	1.07 (0.89-1.28)	1.00 (ref)	0.84 (0.78-0.90)	0.79 (0.72-0.87)	0.68 (0.60-0.78)	0.88 (0.86-0.91)	<0.001
NYUWHS	5619	1.06 (0.88-1.27)	1.00 (ref)	0.85 (0.79-0.91)	0.78 (0.71-0.87)	0.69 (0.61-0.79)	0.89 (0.86-0.91)	<0.001
RERF (c)	-	-	-	-	-	-	-	-
SCHS	5689	1.08 (0.90-1.30)	1.00 (ref)	0.85 (0.79-0.90)	0.79 (0.71-0.87)	0.68 (0.60-0.78)	0.88 (0.86-0.91)	<0.001
SIS	5458	1.08 (0.89-1.29)	1.00 (ref)	0.84 (0.79-0.90)	0.77 (0.69-0.85)	0.68 (0.59-0.78)	0.88 (0.85-0.91)	<0.001
SMC	5582	1.06 (0.89-1.28)	1.00 (ref)	0.85 (0.80-0.91)	0.79 (0.71-0.87)	0.69 (0.60-0.79)	0.89 (0.86-0.91)	<0.001
USRTC	5627	1.03 (0.86-1.25)	1.00 (ref)	0.83 (0.78-0.89)	0.78 (0.71-0.86)	0.68 (0.59-0.77)	0.88 (0.85-0.91)	<0.001
WLHS	5589	1.07 (0.89-1.29)	1.00 (ref)	0.85 (0.79-0.90)	0.79 (0.71-0.87)	0.68 (0.59-0.78)	0.88 (0.86-0.91)	<0.001

(a) HRs adjusted for attained age, cohort, year of birth, age at menarche, age at first birth, number of births, time since last birth and family history of breast cancer
(b) Linear trend per 5 unit difference fitted across BMI values from 18.5 to 49.9 kg/m^2
(c) Insufficient number of cases with BMI at age 45-54 years for this cohort

© 2018 American Medical Association. All rights reserved.

eFigure 1: Relative risk of premenopausal breast cancer in relation to BMI relative to the reference category of 20 kg/m², by age at BMI

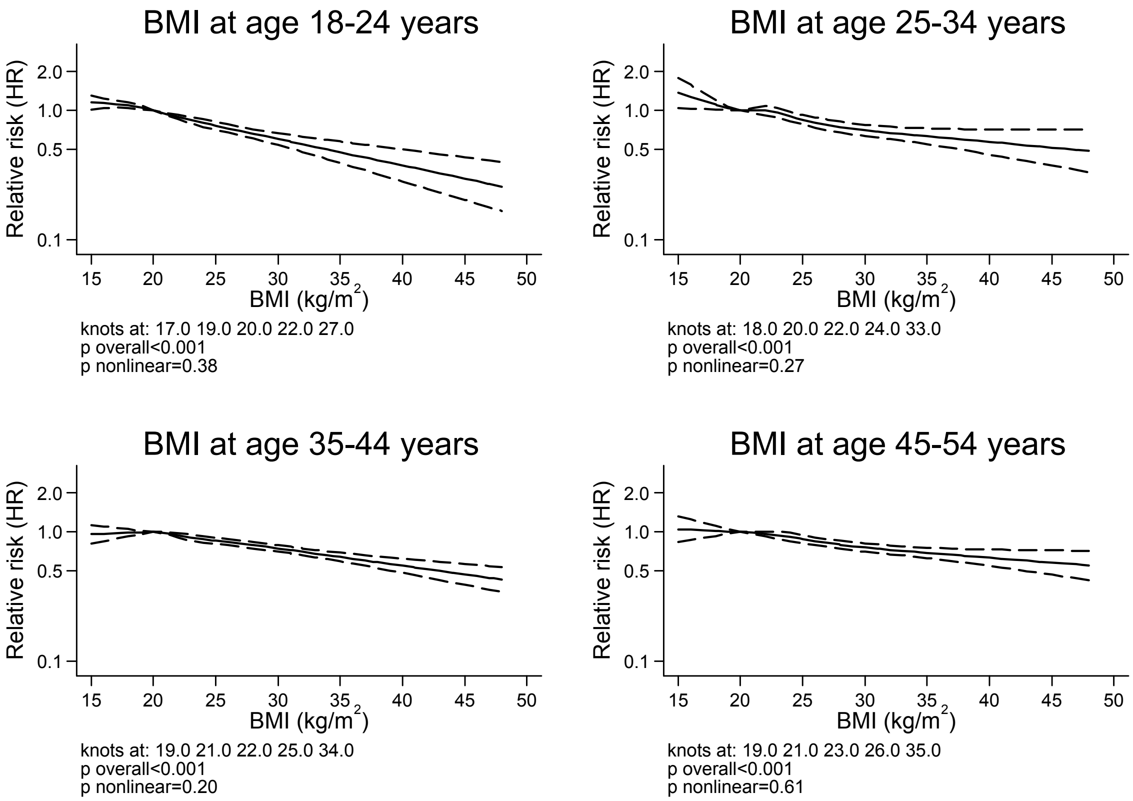

Abbreviations: BMI, Body-mass Index; HR, hazard ratio

HRs obtained from 5-knot restricted cubic spline model adjusted for attained age, cohort, year of birth, age at menarche, age at first birth, number of births, time since last birth and family history of breast cancer. Knot locations are based on Harrell's recommended percentiles[5] as specified in Stata[4], corresponding to the 5th, 25th, 50th, 75% and 95% percentile distribution. Solid line represents hazard ratio relative to the reference group of 20 kg/m², dashed line represents 95% confidence interval of hazard ratio.

© 2018 American Medical Association. All rights reserved.

eFigure 2: Relative risk of premenopausal breast cancer in relation to BMI at ages 18-24 years relative to the reference category of 20 kg/m², by combined ER/PR status of breast cancer

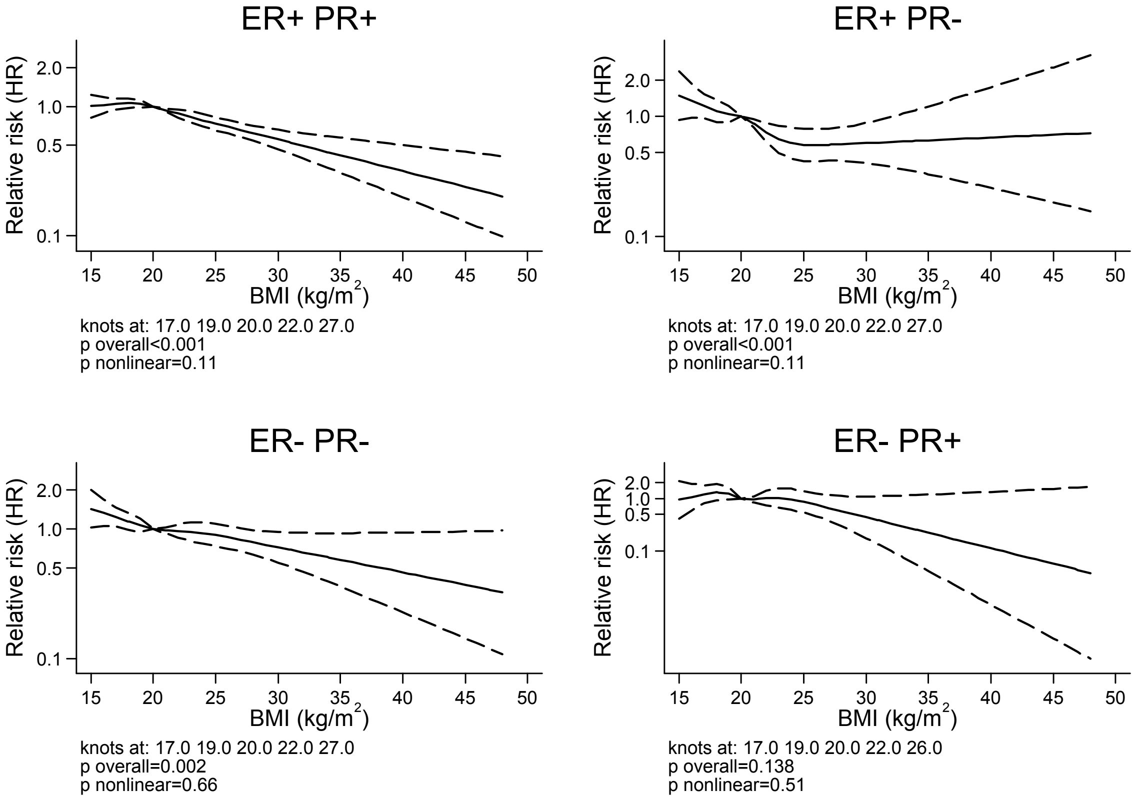

Abbreviations: BMI, Body-mass Index; ER, oestrogen-receptor; HR, hazard ratio; PR, progesterone-receptor

HRs obtained from 5-knot restricted cubic spline model adjusted for attained age, cohort, year of birth, age at menarche, age at first birth, number of births, time since last birth and family history of breast cancer. Knot locations are based on Harrell's recommended percentiles[5] as specified in Stata[4], corresponding to the 5th, 25th, 50th, 75% and 95% percentile distribution. Solid line represents hazard ratio relative to the reference group of 20 kg/m², dashed line represents 95% confidence interval of hazard ratio. Note the different Y-axis for the ER-PR+ category.

© 2018 American Medical Association. All rights reserved.

eFigure 3a: Forest plot of study-specific hazard ratios of premenopausal breast cancer by BMI category, relative to BMI 18.5-24.9 kg/m^2, at age 18-24 years.

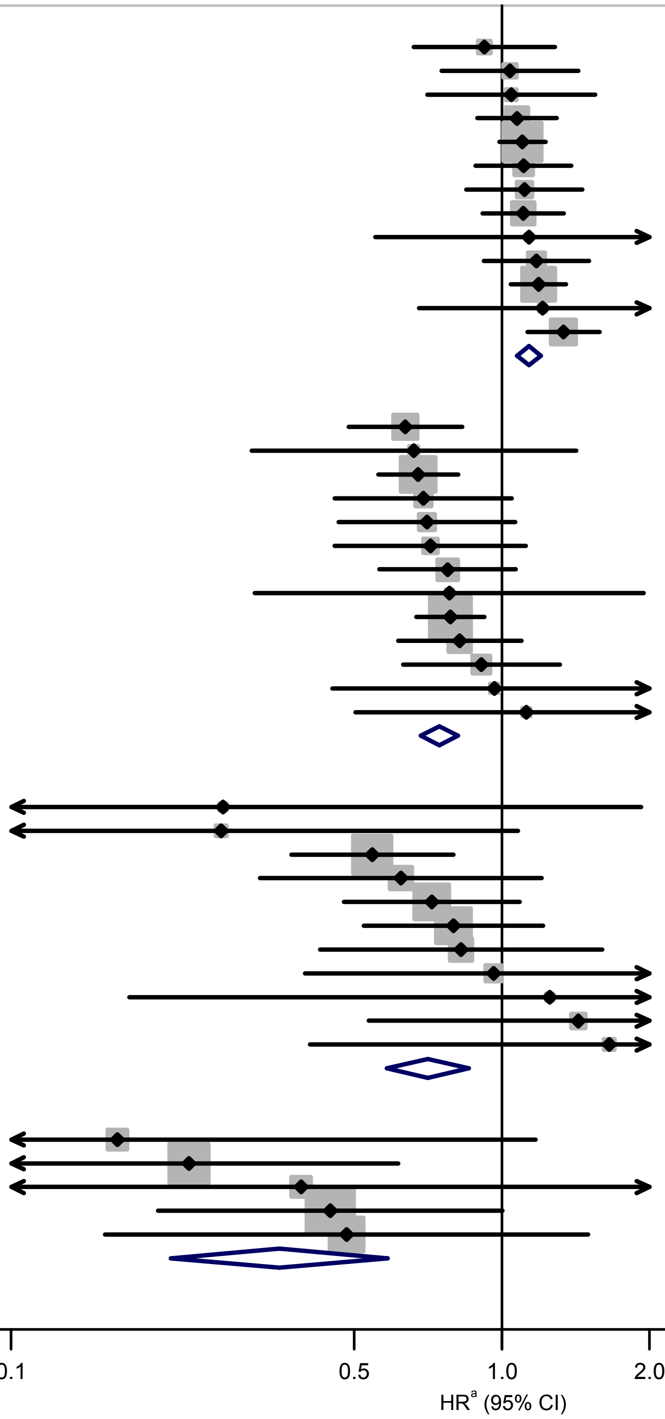

BMI & Study	Cases/ Subjects	Cases/Subjects (reference group)	HR[a] (95% CI)
BMI 15.0-18.4 kg/m^2			
EPIC	40 / 4961	343 / 38235	0.92 (0.66-1.28)
SMC	50 / 3461	152 / 11530	1.04 (0.75-1.43)
CSDLH	30 / 155	174 / 880	1.04 (0.70-1.55)
NOWAC	138 / 11919	595 / 56564	1.07 (0.89-1.29)
NHS2	392 / 11751	1905 / 62062	1.10 (0.99-1.23)
CTS	92 / 5751	493 / 34651	1.11 (0.88-1.39)
GS	58 / 4510	523 / 46854	1.11 (0.85-1.46)
WLHS	140 / 8926	471 / 33153	1.11 (0.91-1.34)
CLUE2	9 / 482	48 / 3025	1.13 (0.55-2.33)
USRTC	79 / 6032	351 / 30844	1.18 (0.92-1.50)
NHS	272 / 8948	1562 / 59671	1.19 (1.04-1.35)
MCC	14 / 1105	64 / 6151	1.21 (0.68-2.17)
BWHS	183 / 7727	547 / 29039	1.34 (1.13-1.58)
Subtotal (I-squared = 0.0%, p = 0.862)			1.14 (1.10-1.20)
BMI 25.0-29.9 kg/m^2			
BWHS	61 / 5166	547 / 29039	0.64 (0.49-0.83)
CSDLH	7 / 52	174 / 880	0.66 (0.31-1.42)
NHS	117 / 6501	1562 / 59671	0.68 (0.56-0.82)
EPIC	24 / 4081	343 / 38235	0.69 (0.46-1.05)
USRTC	24 / 2815	351 / 30844	0.70 (0.46-1.07)
WLHS	20 / 1951	471 / 33153	0.71 (0.46-1.12)
CTS	41 / 3575	493 / 34651	0.77 (0.56-1.07)
MCC	5 / 633	64 / 6151	0.78 (0.31-1.95)
NHS2	165 / 6815	1905 / 62062	0.79 (0.67-0.92)
GS	51 / 6568	523 / 46854	0.82 (0.61-1.10)
NOWAC	30 / 3140	595 / 56564	0.91 (0.63-1.31)
SMC	7 / 549	152 / 11530	0.97 (0.45-2.07)
CLUE2	7 / 402	48 / 3025	1.12 (0.50-2.50)
Subtotal (I-squared = 0.0%, p = 0.906)			0.75 (0.68-0.81)
BMI 30.0-34.9 kg/m^2			
WLHS	1 / 233	471 / 33153	0.27 (0.04-1.92)
USRTC	2 / 618	351 / 30844	0.27 (0.07-1.08)
NHS2	27 / 1546	1905 / 62062	0.54 (0.37-0.80)
CTS	9 / 958	493 / 34651	0.62 (0.32-1.20)
NHS	23 / 1209	1562 / 59671	0.72 (0.48-1.09)
BWHS	23 / 1687	547 / 29039	0.80 (0.52-1.21)
GS	9 / 1349	523 / 46854	0.83 (0.43-1.60)
EPIC	5 / 596	343 / 38235	0.96 (0.40-2.33)
SMC	1 / 65	152 / 11530	1.25 (0.17-9.02)
NOWAC	4 / 275	595 / 56564	1.43 (0.54-3.84)
CSDLH	2 / 10	174 / 880	1.66 (0.41-6.75)
Subtotal (I-squared = 0.0%, p = 0.491)			0.71 (0.58-0.86)
BMI 35.0-49.9 kg/m^2			
CTS	1 / 392	493 / 34651	0.16 (0.02-1.17)
NHS2	4 / 529	1905 / 62062	0.23 (0.09-0.61)
USRTC	1 / 210	351 / 30844	0.39 (0.05-2.78)
BWHS	6 / 863	547 / 29039	0.45 (0.20-1.00)
NHS	3 / 221	1562 / 59671	0.48 (0.16-1.50)
Subtotal (I-squared = 0.0%, p = 0.747)			0.35 (0.21-0.59)

Abbreviations: BMI, Body-mass Index; CI, confidence interval; HR, hazard ratio; for cohort abbreviations see page 3.
[a]HRs adjusted for attained age, year of birth, age at menarche, age at first birth, number of births, time since last birth and family history of breast cancer.

© 2018 American Medical Association. All rights reserved.

eFigure 3b: Forest plot of study-specific hazard ratios of premenopausal breast cancer by BMI category, relative to BMI 18.5-24.9 kg/m², at age 25-34 years.

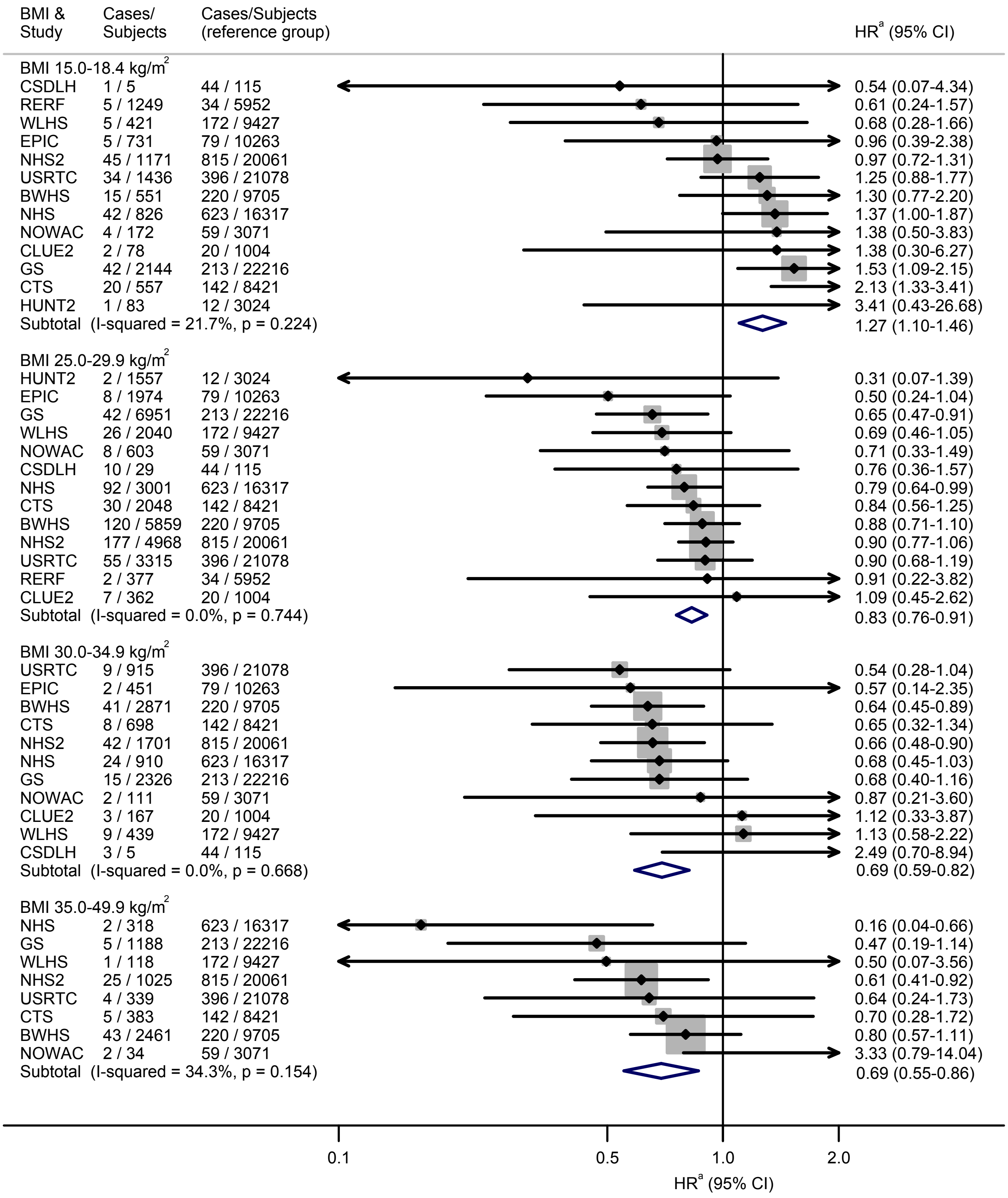

Abbreviations: BMI, Body-mass Index; CI, confidence interval; HR, hazard ratio; for cohort abbreviations see page 3.
[a]HRs adjusted for attained age, year of birth, age at menarche, age at first birth, number of births, time since last birth and family history of breast cancer.

© 2018 American Medical Association. All rights reserved.

eFigure 3c: Forest plot of study-specific hazard ratios of premenopausal breast cancer by BMI category, relative to BMI 18.5-24.9 kg/m^2, at age 35-44 years.

BMI & Study	Cases/ Subjects	Cases/Subjects (reference group)	HR[a] (95% CI)
**BMI 15.0-18.4 kg/m^2**			
GS	5 / 695	373 / 28855	0.60 (0.25-1.45)
SIS	6 / 364	250 / 9514	0.63 (0.28-1.41)
BWHS	5 / 365	265 / 11932	0.63 (0.26-1.52)
EPIC	10 / 850	475 / 29048	0.70 (0.37-1.31)
SMC	4 / 307	143 / 9781	0.88 (0.32-2.38)
RERF	5 / 738	34 / 5158	0.92 (0.36-2.36)
NOWAC	22 / 1596	496 / 34393	0.93 (0.61-1.43)
CSDLH	4 / 16	104 / 394	0.94 (0.33-2.67)
E3N	54 / 1814	710 / 25259	1.07 (0.81-1.41)
NHS	62 / 1781	1495 / 46668	1.09 (0.84-1.40)
HUNT2	1 / 54	56 / 3505	1.13 (0.16-8.24)
USRTC	21 / 1099	462 / 28372	1.20 (0.77-1.85)
WLHS	13 / 639	304 / 20344	1.24 (0.71-2.17)
NHS2	81 / 1983	1607 / 51367	1.32 (1.05-1.65)
CTS	16 / 712	255 / 14816	1.33 (0.80-2.21)
NYUWHS	9 / 120	134 / 2874	1.50 (0.76-2.96)
Subtotal (I-squared = 0.0%, p = 0.644)			1.10 (0.99-1.24)
**BMI 25.0-29.9 kg/m^2**			
HUNT2	20 / 2070	56 / 3505	0.63 (0.38-1.06)
RERF	4 / 854	34 / 5158	0.75 (0.27-2.11)
CSDLH	19 / 102	104 / 394	0.77 (0.47-1.26)
WLHS	66 / 6028	304 / 20344	0.77 (0.59-1.01)
E3N	65 / 2968	710 / 25259	0.78 (0.61-1.01)
NOWAC	83 / 7522	496 / 34393	0.78 (0.62-0.98)
EPIC	150 / 12643	475 / 29048	0.78 (0.65-0.94)
CLUE2	11 / 676	30 / 1506	0.79 (0.39-1.58)
USRTC	107 / 8396	462 / 28372	0.80 (0.65-0.99)
NHS	314 / 12092	1495 / 46668	0.82 (0.73-0.93)
BWHS	205 / 11150	265 / 11932	0.84 (0.70-1.01)
NYUWHS	30 / 834	134 / 2874	0.85 (0.57-1.26)
GS	133 / 11782	373 / 28855	0.87 (0.71-1.06)
CTS	71 / 4687	255 / 14816	0.90 (0.69-1.17)
NHS2	480 / 17545	1607 / 51367	0.90 (0.81-1.00)
MCC	12 / 1027	29 / 2098	0.91 (0.46-1.79)
SMC	37 / 2698	143 / 9781	0.97 (0.67-1.39)
SIS	93 / 3201	250 / 9514	0.99 (0.78-1.26)
Subtotal (I-squared = 0.0%, p = 0.972)			0.85 (0.80-0.89)
**BMI 30.0-34.9 kg/m^2**			
HUNT2	3 / 608	56 / 3505	0.34 (0.11-1.10)
NOWAC	11 / 1579	496 / 34393	0.48 (0.26-0.88)
E3N	7 / 498	710 / 25259	0.49 (0.23-1.03)
GS	34 / 4011	373 / 28855	0.66 (0.46-0.93)
USRTC	32 / 3059	462 / 28372	0.67 (0.47-0.96)
EPIC	41 / 3830	475 / 29048	0.74 (0.54-1.03)
NHS	89 / 3733	1495 / 46668	0.76 (0.61-0.94)
CTS	25 / 1919	255 / 14816	0.77 (0.51-1.17)
NHS2	171 / 6939	1607 / 51367	0.82 (0.70-0.96)
WLHS	16 / 1377	304 / 20344	0.85 (0.52-1.42)
SIS	36 / 1277	250 / 9514	0.90 (0.63-1.28)
CLUE2	6 / 357	30 / 1506	0.91 (0.38-2.22)
CSDLH	7 / 28	104 / 394	0.92 (0.42-2.00)
BWHS	128 / 6378	265 / 11932	0.94 (0.76-1.16)
SMC	8 / 635	143 / 9781	0.94 (0.46-1.92)
MCC	4 / 353	29 / 2098	0.97 (0.33-2.81)
NYUWHS	13 / 284	134 / 2874	1.12 (0.63-2.00)
Subtotal (I-squared = 0.0%, p = 0.630)			0.80 (0.73-0.87)
**BMI 35.0-49.9 kg/m^2**			
NYUWHS	1 / 147	134 / 2874	0.16 (0.02-1.17)
CLUE2	1 / 275	30 / 1506	0.23 (0.03-1.69)
E3N	1 / 135	710 / 25259	0.25 (0.04-1.78)
SIS	12 / 905	250 / 9514	0.43 (0.24-0.77)
NHS	26 / 1552	1495 / 46668	0.51 (0.35-0.76)
USRTC	13 / 1463	462 / 28372	0.56 (0.32-0.97)
NHS2	82 / 4694	1607 / 51367	0.57 (0.46-0.71)
GS	16 / 1941	373 / 28855	0.63 (0.38-1.05)
BWHS	74 / 5536	265 / 11932	0.65 (0.50-0.84)
EPIC	17 / 1466	475 / 29048	0.81 (0.49-1.31)
SMC	2 / 151	143 / 9781	0.93 (0.23-3.75)
WLHS	6 / 446	304 / 20344	0.95 (0.42-2.14)
CTS	21 / 1236	255 / 14816	1.03 (0.66-1.62)
NOWAC	6 / 382	496 / 34393	1.04 (0.46-2.34)
Subtotal (I-squared = 17.6%, p = 0.262)			0.63 (0.55-0.71)

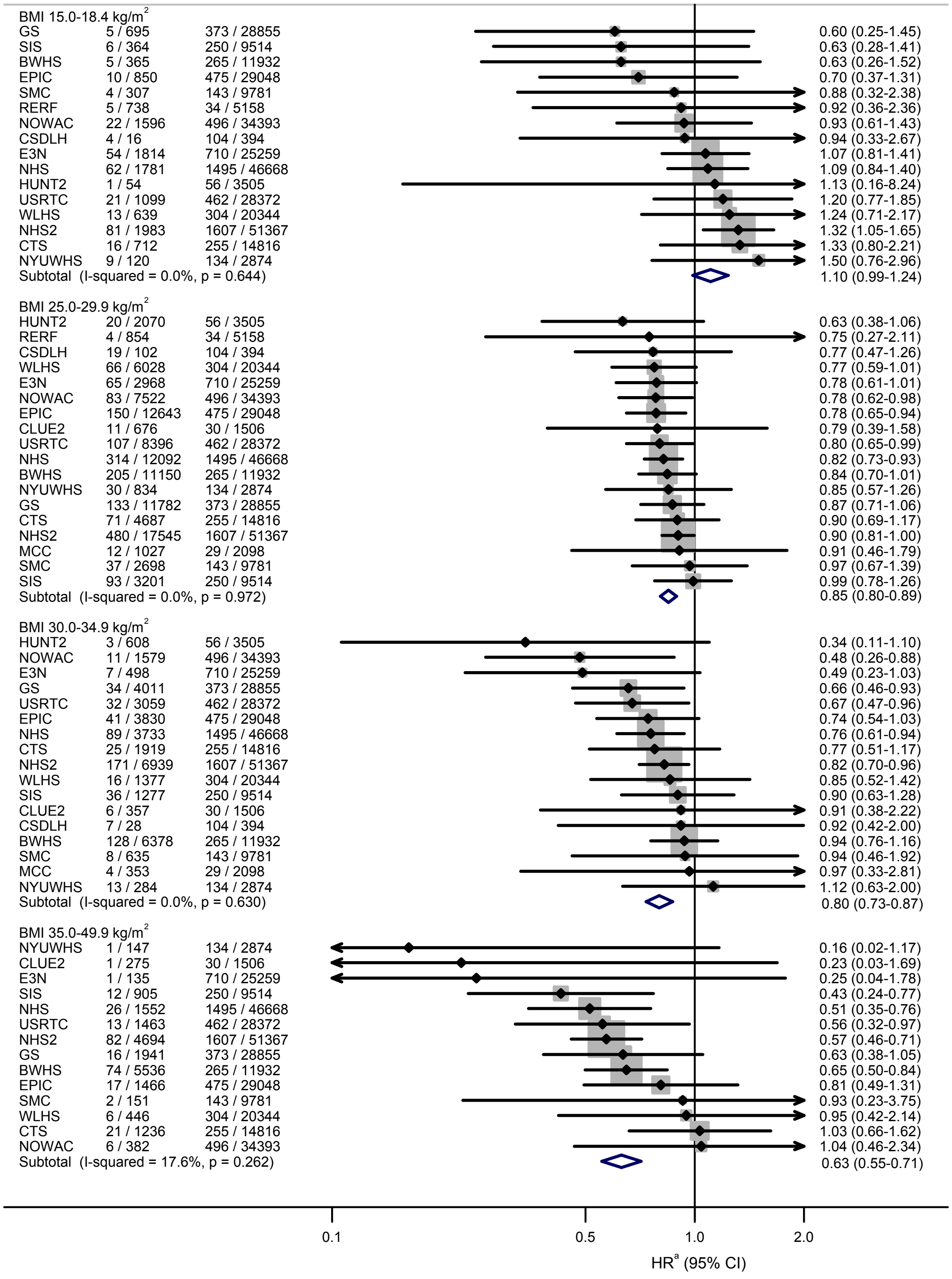

Abbreviations: BMI, Body-mass Index; CI, confidence interval; HR, hazard ratio; for cohort abbreviations see page 3.
[a]HRs adjusted for attained age, year of birth, age at menarche, age at first birth, number of births, time since last birth and family history of breast cancer.

© 2018 American Medical Association. All rights reserved.

eFigure 3d: Forest plot of study-specific hazard ratios of premenopausal breast cancer by BMI category, relative to BMI 18.5-24.9 kg/m², at age 45-54 years.

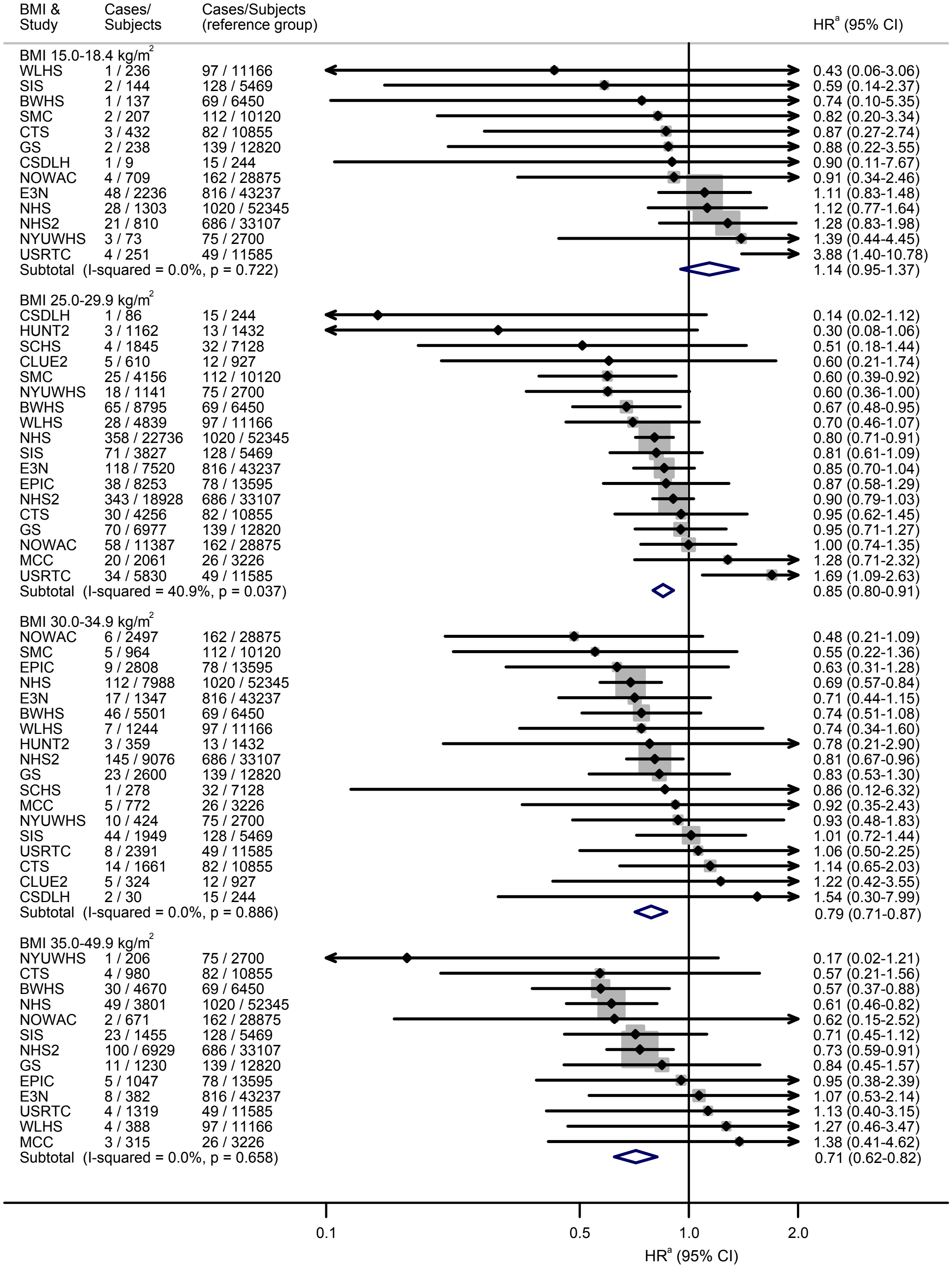

Abbreviations: BMI, Body-mass Index; CI, confidence interval; HR, hazard ratio; for cohort abbreviations see page 3.
[a]HRs adjusted for attained age, year of birth, age at menarche, age at first birth, number of births, time since last birth and family history of breast cancer.

© 2018 American Medical Association. All rights reserved.

eFigure 4: Relative risk of premenopausal breast cancer per 5 kg/m² difference in BMI, by age at BMI and attained age during follow-up

Age at BMI and attained age, yrs	No. of cases	HR[a] per 5 kg/m² (95% CI)
**BMI at age 18-24 years**		
<40	799	0.83 (0.73-0.93)
40-44	1968	0.78 (0.71-0.85)
45-49	3711	0.75 (0.71-0.80)
50-54	1430	0.74 (0.66-0.82)
		P_{int} =0.64
**BMI at age 25-34 years**		
<40	828	0.86 (0.79-0.93)
40-44	1127	0.87 (0.81-0.94)
45-49	1245	0.81 (0.75-0.88)
50-54	454	0.89 (0.79-1.01)
		P_{int} =0.23
**BMI at age 35-44 years**		
<40	563	0.87 (0.79-0.95)
40-44	2731	0.90 (0.86-0.94)
45-49	4881	0.87 (0.84-0.90)
50-54	1856	0.82 (0.77-0.87)
		P_{int} =0.13
**BMI at age 45-54 years**		
45-49	3239	0.90 (0.86-0.93)
50-54	2367	0.86 (0.83-0.91)
		P_{int} =0.84

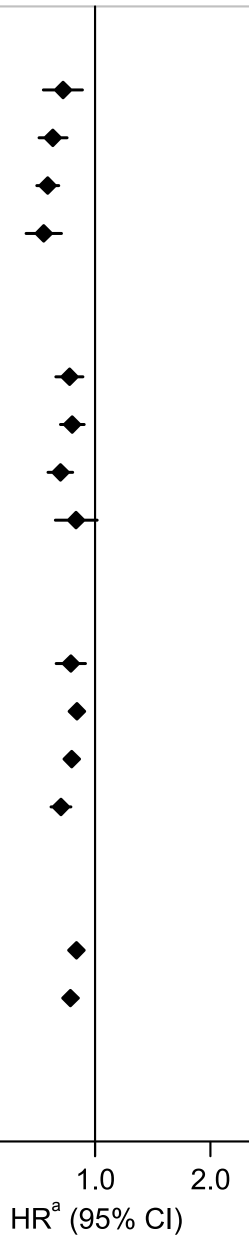

Abbreviations: BMI, body-mass index; CI, confidence interval; HR, hazard ratio; P_{int}=p value for interaction

[a]HRs represent linear trend per 5 kg/m² difference in BMI from 18.5 to 49.9 kg/m² and are adjusted for attained age, cohort and year of birth, age at menarche, age at first birth, number of births, time since last birth and family history of breast cancer

© 2018 American Medical Association. All rights reserved.

eFigure 5: Relative risk of premenopausal breast cancer per 5 kg/m^2 difference in BMI, by age at BMI and combined oestrogen and progesterone receptor status of breast cancer

Age at BMI and hormone receptor status	No. of cases	HR[a] per 5 kg/m^2 (95% CI)
BMI at age 18-24 years		
ER+PR+	2910	0.76 (0.70-0.81)
ER+PR-	467	0.70 (0.57-0.86)
ER-PR+	205	0.63 (0.48-0.82)
ER-PR-	971	0.85 (0.76-0.95)
		P_{int} =0.08
BMI at age 25-34 years		
ER+PR+	1432	0.84 (0.79-0.90)
ER+PR-	210	0.91 (0.77-1.07)
ER-PR+	97	0.62 (0.48-0.80)
ER-PR-	472	0.95 (0.86-1.04)
		P_{int} =0.01
BMI at age 35-44 years		
ER+PR+	3772	0.87 (0.84-0.91)
ER+PR-	602	0.79 (0.71-0.87)
ER-PR+	302	0.67 (0.58-0.78)
ER-PR-	1169	0.97 (0.91-1.02)
		P_{int} <0.001
BMI at age 45-54 years		
ER+PR+	2356	0.91 (0.88-0.95)
ER+PR-	406	0.75 (0.67-0.84)
ER-PR+	185	0.81 (0.69-0.96)
ER-PR-	631	0.94 (0.87-1.01)
		P_{int} = 0.005

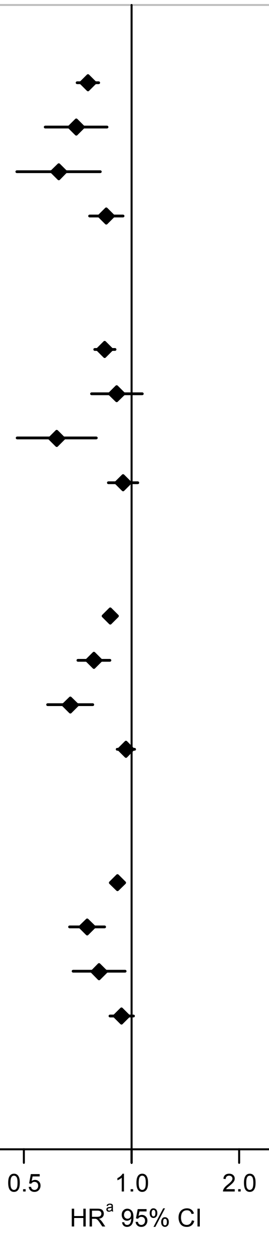

Abbreviations: BMI, body-mass index; CI, confidence interval; ER, oestrogen-receptor; HR, hazard ratio; PR, progesterone-receptor; P_{int}=p value for interaction

[a]HRs represent linear trend per 5 kg/m^2 difference in BMI from 18.5 to 49.9 kg/m^2 and are adjusted for attained age, cohort, year of birth, age at menarche, age at first birth, number of births, time since last birth and family history of breast cancer. Tests for heterogeneity in effect by combined ER/PR status was obtained from an Augmentation model including all four endpoints.[8]

© 2018 American Medical Association. All rights reserved.

eFigure 6: Relative risk of premenopausal breast cancer per 5 kg/m^2 difference in BMI at age 18-24 years, by selected other breast cancer risk factors

Risk factor	No. of cases	HR[a] per 5 kg/m^2 (95% CI)
**Age at menarche, years**		
<12	1806	0.78 (0.72, 0.84)
12-13	4386	0.77 (0.72, 0.81)
14+	1562	0.75 (0.67, 0.84)
		P_{int} =0.89
**Adult height, cms**		
<160	1378	0.79 (0.71, 0.87)
160-169	4378	0.76 (0.71, 0.80)
170+	2152	0.79 (0.72, 0.85)
		P_{int} =0.65
**Ever had birth**		
No	1200	0.72 (0.66, 0.79)
Yes	6461	0.78 (0.74, 0.82)
		P_{int} =0.16
**Number of births**		
1	1160	0.82 (0.74, 0.90)
2	3061	0.79 (0.74, 0.86)
3+	2191	0.72 (0.65, 0.79)
		P_{int} =0.12
**Age at first birth, years**		
<25	2472	0.77 (0.71, 0.83)
25-34	3550	0.78 (0.73, 0.83)
35+	383	0.81 (0.67, 0.97)
		P_{int} =0.89
**Time since last birth, years**		
0-4	298	0.89 (0.72, 1.09)
5-9	814	0.84 (0.74, 0.96)
10-14	1431	0.75 (0.68, 0.84)
15-19	1715	0.76 (0.69, 0.84)
20+	1691	0.77 (0.70, 0.85)
		P_{int} =0.66
**Ever used oral contraception**		
No	1267	0.68 (0.61, 0.76)
Yes	6045	0.79 (0.75, 0.83)
		P_{int} =0.02
**Ever diagnosis with PCOS**		
No	1015	0.78 (0.69, 0.90)
Yes	217	0.67 (0.53, 0.86)
		P_{int} =0.28
**Family history of breast cancer**		
No	5889	0.75 (0.72, 0.79)
Yes	1610	0.80 (0.73, 0.87)
		P_{int} =0.32
**Ever mammogram**		
No	1322	0.84 (0.75, 0.93)
Yes	3706	0.76 (0.71, 0.81)
		P_{int} =0.12
**Race/ethnicity**		
Black	709	0.84 (0.76, 0.93)
Asian	79	0.69 (0.40, 1.18)
White	5922	0.73 (0.70, 0.77)
		P_{int} =0.08
**Year of birth**		
1900-1939	976	0.75 (0.66, 0.85)
1940-1949	1770	0.72 (0.65, 0.79)
1950-1959	3623	0.78 (0.73, 0.83)
1960+	1539	0.80 (0.73, 0.87)
		P_{int} =0.41

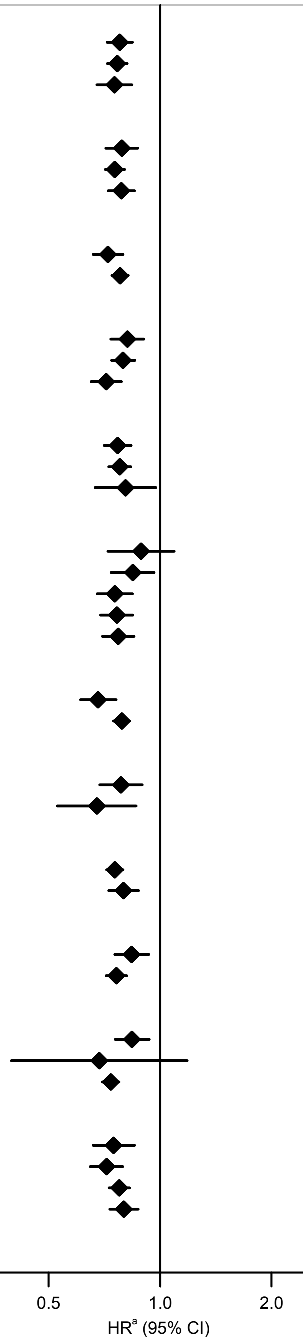

Abbreviations: BMI, body-mass index; CI, confidence interval; HR, hazard ratio; PCOS, polycystic ovary syndrome; P_{int}=p value for interaction

[a]HRs represent linear trend per 5 kg/m^2 difference in BMI from 18.5 to 49.9 kg/m^2. HRs adjusted for attained age, cohort, year of birth, age at menarche, age at first birth, number of births, time since last birth and family history of breast cancer. Stratifying risk factors are time-updated, where data from follow-up questionnaires have been provided.

© 2018 American Medical Association. All rights reserved.

References

1. Nichols HB, Schoemaker MJ, Wright LB, et al. The Premenopausal Breast Cancer Collaboration: A Pooling Project of Studies Participating in the National Cancer Institute Cohort Consortium. *Cancer Epidemiol Biomarkers Prev.* Sep 2017;26(9):1360-1369.
2. WHO. WHO Expert Consultation: Appropriate body-mass index for Asian populations and its implications for policy and intervention strategies. *Lancet.* Jan 10 2004;363(9403):157-163.
3. Goldhirsch A, Winer EP, Coates AS, et al. Personalizing the treatment of women with early breast cancer: highlights of the St Gallen International Expert Consensus on the Primary Therapy of Early Breast Cancer 2013. *Ann Oncol.* Sep 2013;24(9):2206-2223.
4. StataCorp, ed *Stata Statistical Software: Release 14.* College Station, Texas: StataCorp LP; 2015.
5. Harrell F. *Regression Modeling Strategies: With Applications to Linear Models, Logistic Regression, and Survival Analysis.* New York: Springer; 2001.
6. Cox DR. Regression Models and Life-Tables. *Journal of the Royal Statistical Society.* 1972;34(2):187-220.
7. Barlow WE, Ichikawa L, Rosner D, Izumi S. Analysis of case-cohort designs. *J Clin Epidemiol.* Dec 1999;52(12):1165-1172.
8. Lunn M, McNeil D. Applying Cox regression to competing risks. *Biometrics.* Jun 1995;51(2):524-532.

© 2018 American Medical Association. All rights reserved.
